# A global core outcome measurement set for snakebite clinical trials

**DOI:** 10.1016/S2214-109X(22)00479-X

**Published:** 2023-02-01

**Authors:** Michael Abouyannis, Hanif Esmail, Mainga Hamaluba, Mwanajuma Ngama, Hope Mwangudzah, Noni Mumba, Betty K Yeri, Salim Mwalukore, Hassan J Alphan, Dinesh Aggarwal, Gabriel Alcoba, Nick Cammack, Jean-Philippe Chippaux, Matthew E Coldiron, José M Gutiérrez, Abdulrazaq G Habib, Robert A Harrison, Geoffrey K Isbister, Eric J Lavonas, Diogo Martins, Isabela Ribeiro, James A Watson, David J Williams, Nicholas R Casewell, Sarah A Walker, David G Lalloo

**Affiliations:** Centre for Snakebite Research and Interventions, Liverpool School of Tropical Medicine, Liverpool, UK; KEMRI–Wellcome Research Programme, Kilifi, Kenya; MRC Clinical Trials Unit at UCL, London, UK; Institute for Global Health, University College London, London, UK; KEMRI–Wellcome Research Programme, Kilifi, Kenyam; Centre for Tropical Medicine & Global Health, Nuffield Department of Medicine, Oxford, UK; KEMRI–Wellcome Research Programme, Kilifi, Kenya; KEMRI–Wellcome Research Programme, Kilifi, Kenya; Department of Medicine, University of Cambridge, UK; Medical Department, Médecins Sans Frontières/Doctors Without Borders, Geneva, Switzerland; Division of Tropical and Humanitarian Medicine, Geneva University Hospitals, Geneva, Switzerland; Wellcome Trust, London, UK; University of Paris Cité, French National Reseach Institute For Sustainable Development, Monther and child in the tropics: pathogens, health system, and epidemiological transformation unit, Paris, France; Epicentre, Paris, France; Instituto Clodomiro Picado, Facultad de Microbiología, Universidad de Costa Rica, San José, Costa Rica; Bayero University Department of Infectious and Tropical Diseases, Kano, Nigeria; Centre for Snakebite Research and Interventions, Liverpool School of Tropical Medicine, Liverpool, UK; Clinical Toxicology Research Group, University of Newcastle, Newcastle, NSW, Australia; Department of Emergency Medicine, Denver Health and Hospital Authority, Denver, Colorado, USA and Department of Emergency Medicine, University of Colorado School of Medicine, Aurora, Colorado, USA; Wellcome Trust, London, UK; Drugs for Neglected Diseases Initiative, Geneva, Switzerland; Centre for Tropical Medicine & Global Health, Nuffield Department of Medicine, Oxford, UK; Mahidol Oxford Research Unit, Faculty of Tropical Medicine, Mahidol University, Bangkok, Thailand; Regulation and Prequalification Department, Access to Medicines and Health Products Division, World Health Organization, Geneva, Switzerland; Centre for Snakebite Research and Interventions, Liverpool School of Tropical Medicine, Liverpool, UK; MRC Clinical Trials Unit at UCL, London, UK; Centre for Snakebite Research and Interventions, Liverpool School of Tropical Medicine, Liverpool, UK

## Abstract

Snakebite clinical trials have often used heterogeneous outcome measures and there is an urgent need for standardisation. A globally representative group of key stakeholders came together to reach consensus on a globally relevant set of core outcome measurements. Outcome domains and outcome measurement instruments were identified through searching the literature and a systematic review of snakebite clinical trials. Outcome domains were shortlisted by use of a questionnaire and consensus was reached among stakeholders and the patient group through facilitated discussions and voting. Five universal core outcome measures should be included in all future snakebite clinical trials—mortality, WHO disability assessment scale, patient-specific functional scale, acute allergic reaction by Brown criteria, and serum sickness by formal criteria. Additional syndrome-specific core outcome measures should be used depending on the biting species. This core outcome measurement set provides global standardisation, supports the priorities of patients and clinicians, enables meta-analysis, and is appropriate for use in low-income and middle-income settings.

## Introduction

As articulated by Kofi Annan, “Snakebite is the biggest public health crisis you have likely never heard of.”^1^ Snakebites cause an estimated 1·8 million envenomings and 94 000 deaths each year, with the highest burden in Asia and sub-Saharan Africa.^2^ On June 9, 2017, the WHO classified snakebite as a priority neglected tropical disease, and since then increased funding has been allocated by major donors. The need to standardise outcome measure reporting is crucial, particularly given the pipeline of snakebite therapeutics with multispecies specificity, which will require clinical trial assessment across multiple regions.^3,4^ Standardisation of reporting of allergic reactions to antivenom products is essential.

A core outcome measurement set provides a list of outcome measures to be reported in all clinical trials in a disease area.^5^ The core outcome measurement set does not preclude trialists from selecting primary, secondary, or safety outcome measures that are not in the core set. Snakebite presents a unique challenge as different snake species cause diverse clinical syndromes.

In preparation for developing the core outcome measurement set, a systematic review was done.^6^ Over 40 randomised controlled trials have been undertaken, although heterogeneity has largely prevented meta-analyses.^6^ Many trials have used poorly defined, non-reproducible outcome measures, and only trials in high-income settings have included patient-centred outcome measures.^6^

To address this priority, a globally diverse multidisciplinary stakeholder group adopted a structured evidence-based method to reach consensus on a global set of core outcome measurements for snakebite clinical trials.

## Methods

### Scope of the core outcome measurement set

Ten core criteria for choosing the outcome measurements were identified (figure). (1) To be comprised of clearly defined and reproducible outcome measures. (2) To be adaptable to the global clinical diversity of snakebite envenoming. (3) To be relevant to all regions where snakebite envenoming is endemic, including Africa, the Americas, Asia, Europe, and Oceania. (4) To be feasible for use in low-resource, middle-resource, and high-resource settings. (5) To be acceptable to clinical trial participants. (6) To include patient-centred measures that are endorsed by a patient group. (7) To include at least one clinically relevant core outcome measure for each syndrome of envenoming. (8) When laboratory measures or surrogate markers are included, for these to be appropriately validated as trial outcome measures. (9) To be usable in large-sample-size phase 3 clinical trials, and small-sample-size phase 2 clinical trials. (10) To be usable in clinical trials of antivenom or small molecule therapeutics.

### Participants

The stakeholder group was formed of 19 members and included leading snakebite experts, statisticians, trial methodologists, funders, drug development advisors, and policy makers, with representation from Africa, the Americas, Asia, Europe, and Oceania. This group was formed as a Wellcome Trust-funded initiative to improve the methodology of snakebite clinical trials. Members were selected on the basis of their recent involvement in snakebite clinical trials or relevant clinical trial methodology in the past 10 years. The views of the stakeholder group members were their own, and not of their associated institutes (appendix p 4). A separate expert group was formed to enable broader input from the snakebite academic community. All corresponding authors of the 58 studies identified through our systematic review were invited, and so were any individuals recommended by the stakeholder group.^6^

The snakebite patient advisory group was formed of 13 adults—ten with direct experience of snakebite, and three parents of children who had experienced snakebite. All participants were from Kilifi County, Kenya, which is a rural region with a high burden of snakebite. The patient advisory group was formed at Kenya Medical Research Institute (KEMRI)–Wellcome Trust in 2021 to enable people who have experienced snakebite to influence future research priorities.

### Shortlisting core outcome domains

Through our systematic review, 153 unique outcome measures were extracted verbatim, and 60 outcome domains were identified.^6^ A shortlisting questionnaire (appendix p 5) was circulated to the stakeholder and expert groups. Domains were scored and categorised as: 7–9 (essential); 6–4 (desirable); or 3–1 (inappropriate). At least two domains were shortlisted for each clinical syndrome of envenoming, based on score rankings. Respondents could recommend additional domains not identified through the systematic review, and these were automatically shortlisted (appendix p 13).

Four universal and seven syndrome-specific categories were agreed by the stakeholder and expert groups: Universal (all snake species) categories include: (1) mortality, (2) disability, (3) acute allergic reaction, and (4) serum sickness; and syndrome-specific (snake species-specific) categories include: (1) neurotoxicity, (2) haemotoxicity, (3) coagulopathy, (4) local tissue damage, (5) renal injury, (6) systemic myotoxicity, and (7) hypotension.

### Reaching consensus on core outcome measures

A primary literature review was prepared by one investigator (MA), circulated to the stakeholder and expert groups for wider input, and maintained as a live document (appendix p 14). This literature review included relevant information on outcome domains and outcome measurement instruments. A series of four stakeholder group consensus meetings were held, with a total duration of 10 h. One investigator (MA) presented a summary of the questionnaire results and literature for each shortlisted outcome domain. Meetings were chaired by a senior investigator (DGL). A semistructured discussion on outcome domains and outcome measurement instruments took place, and all stakeholder group members had the opportunity to share their views. Once all views had been shared, anonymous voting took place with at least 70% agreement needed to confirm a core outcome measure.

### Patient involvement

The snakebite patient advisory group had a semi-structured group discussion facilitated by two clinical officers with experience of conducting snakebite research, and members of the KEMRI–Wellcome community liaison group. Discussion was in Swahili language, including translation of all written materials. There was an open discussion of the members’ experiences of snakebite and what they found most challenging. Subsequently, feedback was sought on the patient-centred outcome measures that were selected by the stakeholder group. Patient-centred outcome measures were to be included as core outcome measures only if they were endorsed by the snakebite patient advisory group.

The study protocol was prospectively registered on the COMET Initiative database.^7^

## Results

### Overview

77 outcome domains were included on the shortlisting questionnaire, and the scorings for these are available (appendix pp 41–45). We provide an overview of the core outcome measurement set (figure) and the full core outcome measurement set is also available (appendix p 48).

### Mortality

All-cause mortality was graded as an essential core outcome domain (appendix p 48). Consensus discussion on the optimal period of follow-up converged on 6 weeks after randomisation as most deaths attributable to envenoming occur within this interval.

### Disability scales

The WHO Disability Assessment Scale (WHODAS) and the Patient-Specific Functional Scale (PSFS) were the highest scoring outcome measurement instruments for grading disability (appendix p 48). The WHODAS has been extensively validated in low-resource settings, it is available in many languages, and it can be adapted for use in children.^8,9^ WHODAS should be measured 6 weeks after randomisation with the 12-item version, which is less burdensome to administer than the 36-item version (appendix p 54). The PSFS has been used in a snakebite clinical trial, it can be administered by telephone, and it is highly patient centred.^10,11^ It complements the WHODAS tool as it is less structured and more patient led. Measurement is at 2 weeks, as per the previous clinical trial, and 6 weeks, to align with other core outcome measures.

The snakebite patient advisory group reviewed the WHODAS and PSFS tools and piloted their translated versions. It was reported that these tools were useful and usable. Four members of the snakebite patient advisory group self-identified as having low literacy or illiteracy but, with support, were able to complete the WHODAS questionnaire. The adaptability of the PSFS was viewed positively and the numeracy requirement of the Likert scale was not seen as a barrier. With endorsement from the snakebite patient advisory group, WHODAS and PSFS were included in the core outcome measurement set.

### Acute allergic reaction

The Brown criteria and National Institute of Allergy and Infectious Diseases definitions of allergic reactions are the only outcome measurement instruments that have been used to define allergic reactions in snakebite clinical trials (appendix p 48).^12,13^ The Brown criteria were selected preferentially as they are sensitive to non-severe allergic reactions such as an isolated skin rash.

### Serum sickness

The only reproducible definition of serum sickness that could be identified was a telephone-delivered questionnaire used in a prospective study in Australia (appendix p 48).^14^ The stakeholder group agreed that this outcome measurement instrument should be included. Arthritis was added to the definition as it is a hallmark of serum sickness.

### Neurotoxicity

Requirement for invasive ventilation and duration of ventilation were scored as essential and were viewed as being highly clinically relevant (appendix p 49). An existing core outcome measurement set^15^ that provides clear definitions of these domains was agreed for inclusion in the tool.

Minor modifications were made to reflect practices in some low-resource settings, including recognising the use of laryngeal mask airways and manual ventilation. Neurotoxic envenoming has a rapid onset and therefore a cutoff time of onset of intubation or ventilation within 48 h of randomisation was agreed to avoid misclassification of intubation and ventilation for other causes. When measuring duration of ventilation, death is a competing risk that would bias toward shorter duration within a treatment group with a higher mortality rate. To avoid this bias, death should be treated as a competing risk in all participants, and a separate analysis should be based on the probability of successful unassisted breathing conditional on remaining alive on ventilation.

### Haemotoxicity

An established definition of major bleeding was scored as essential, has been used in numerous snakebite clinical trials,^6,16^ and was unanimously agreed for inclusion in the core outcome measurement set (appendix p 50).

The European Medicines Agency criteria for clinically relevant non-major bleeding (CRNMB) provide a definition for less severe bleeding events. These criteria are likely to be more responsive to treatment effects than a definition based on major bleeding events.^17,18^ Two definitions of CRNMB were agreed on: (1) proportion with cessation of early bleeding events within 6 h of randomisation and (2) proportion with new bleeding events occurring 6–48 h after randomisation.

### Coagulopathy

The 20-min whole blood clotting test (20WBCT) and laboratory measured international normalised ratio were shortlisted (appendix p 50). The 20WBCT has been used in numerous snakebite clinical trials^6^ and is simple to measure in low-resource settings. There were concerns among some stakeholder and expert group members that 20WBCT has not been validated as a trial outcome measure and might have low sensitivity.

The laboratory-measured international normalised ratio offers a standardised and quantifiable measure of coagulopathy. Although it was agreed among the stakeholder group that international normalised ratio offers a more accurate and better validated outcome measure than 20WBCT, some members held strong views that it is too challenging to implement in low-resource settings. Nevertheless, many snakebite clinical trials done in low-resource settings have included laboratory-based coagulation assays.^6^ Point-of-care international normalised ratio testing is more feasible to implement than laboratory-measured international normalised ratio testing, but it has not been validated for detecting venom-induced consumption coagulopathy.^19^

The stakeholder group’s votes did not reach the threshold for including 20WBCT (22% voted in favour) but did favour including laboratory-measured international normalised ratio (90% voted in favour). Further research on the validity and accuracy of 20WBCT as an outcome measure is needed, and it could be included as a core outcome measure if data supporting its validity emerge.

### Local tissue damage

The proportion of participants requiring surgery to manage local tissue damage was included as, although surgical interventions are infrequent, they are highly clinically significant (appendix pp 50–51).

Ordinal pain scale did not reach the threshold for inclusion with concerns as to its validity for measuring local tissue damage, particularly given geographical variations in the use of analgesics.

Surface area of skin necrosis provides a quantifiable, clinically relevant measure, which is more responsive than the proportion of participants requiring surgery. It was included in the core outcome measurement set and can be recorded with digital imaging technology or manual methods (such as a tape measure).

### Renal injury

The renal injury category is for snake species that cause renal injury (namely *Daboia russelii*) or clinically significant systemic myotoxicity (appendix p 51).

An outcome measure based on the proportion of participants requiring renal replacement therapy (RRT) is clinically relevant, although absence of standardised criteria for commencing the RRT and low availability of it in low-income and middle-income countries (LMIC) are limitations that introduce variability between trial sites. With the proviso that the criteria for commencing RRT are reported, this measure was included as a core outcome.

Short-term changes in renal function (including acute kidney injury) are not necessarily clinically significant, and are not recognised as valid outcome measures by the US Food and Drug Administration.^20^ Conversely, de-novo chronic kidney disease is clinically significant but measuring renal function 3 months after randomisation might be challenging in LMIC settings. A decline in estimated glomerular filtration rate (eGFR) that persists for longer than 30 days is associated with end-stage renal disease, and is included in the Major Adverse Kidney Events trial endpoints.^20,21^ To align with the timepoint of other core outcome measures, and with the threshold from an existing renal endpoint,^22^ the proportion of participants with at least 30% decline in eGFR at 6 weeks after randomisation was agreed as a core outcome measure.

### Systemic myotoxicity

Serum creatine kinase is an established measure of systemic myotoxicity; however, the clinical significance of a rise in creatine kinase that is not associated with nephrotoxicity is uncertain (appendix p 51). A direct measure of systemic myotoxicity was not included and, instead, the renal injury core outcome measures should be used.

### Hypotension

The hypotension category is particularly relevant for envenoming by species such as European *Vipera* spp (appendix p 51). Standardised age-adjusted criteria for hypotensive shock, which were validated in LMIC settings, were identified.^23^ These should be measured 3 h after randomisation, as an effective therapeutic administered to the participant should correct venom-induced hypotension by this time.

## Discussion

We established a globally relevant, evidence-based, patient-centred, core outcome measurement set for snakebite clinical trials. This set will enable meta-analysis, support adoption of clinically meaningful endpoints, and serve the needs of patients. The universal core outcome measures (mortality, WHODAS, PSFS, anaphylaxis, and serum sickness) provide global standardisation for these essential therapeutic indicators. The syndrome-specific core outcome measures allow adaptation for specific biting species. This tool sets the minimum, with there being no limit on other outcome measures that can be included in snakebite trials. This core set will be updated as new evidence emerges. In future iterations we hope to receive a broader input, particularly from patient groups based in Africa and Asia.

Uptake of this set is key, and ease of implementation was carefully considered. Only two follow-up visits are needed (at 2 weeks and 6 weeks), and the measurement instruments (PSFS, WHODAS, and serum sickness assessment) are simple to administer via telephone.^8,11,14^

The needs of LMIC-based researchers were prioritised: long periods of follow-up have been avoided, the WHODAS has been extensively validated in LMIC settings, and the patient group members are representative of a snakebite endemic region. A potential challenge at implementation might be the measurement of international normalised ratio in LMIC settings. Although most of the stakeholder group favoured international normalised ratio over 20WBCT, the financial and logistical challenges of implementing this assay in rural LMIC settings were highlighted. In some scenarios, such as clinical trials with a restricted budget, implementation of laboratory measured international normalised ratio might be impossible, necessitating the use of 20WBCT instead. Importantly, 20WBCT is not necessarily an inaccurate outcome measure, rather data are scarce—two studies have tested the performance of 20WBCT after antivenom and both identified poor sensitivity.^24^ International normalised ratio is only recommended as an outcome measure, and not as an eligibility criterion for enrolment.

Consensus has been reached among major stakeholders to standardise outcome measure reporting in snakebite clinical trials. By approaching snakebite as a global issue, the divide in the standards of trial methodology adopted in high-income and low-income settings can be bridged. WHO have set the ambitious goal of reducing global snakebite disabilities and deaths by 50% by 2030. As improved therapeutics emerge, appropriate reporting of outcome measures will be essential for tracking our progress towards this goal.

## Supplementary Material

Supplementary appendix

## Figures and Tables

**Figure:**
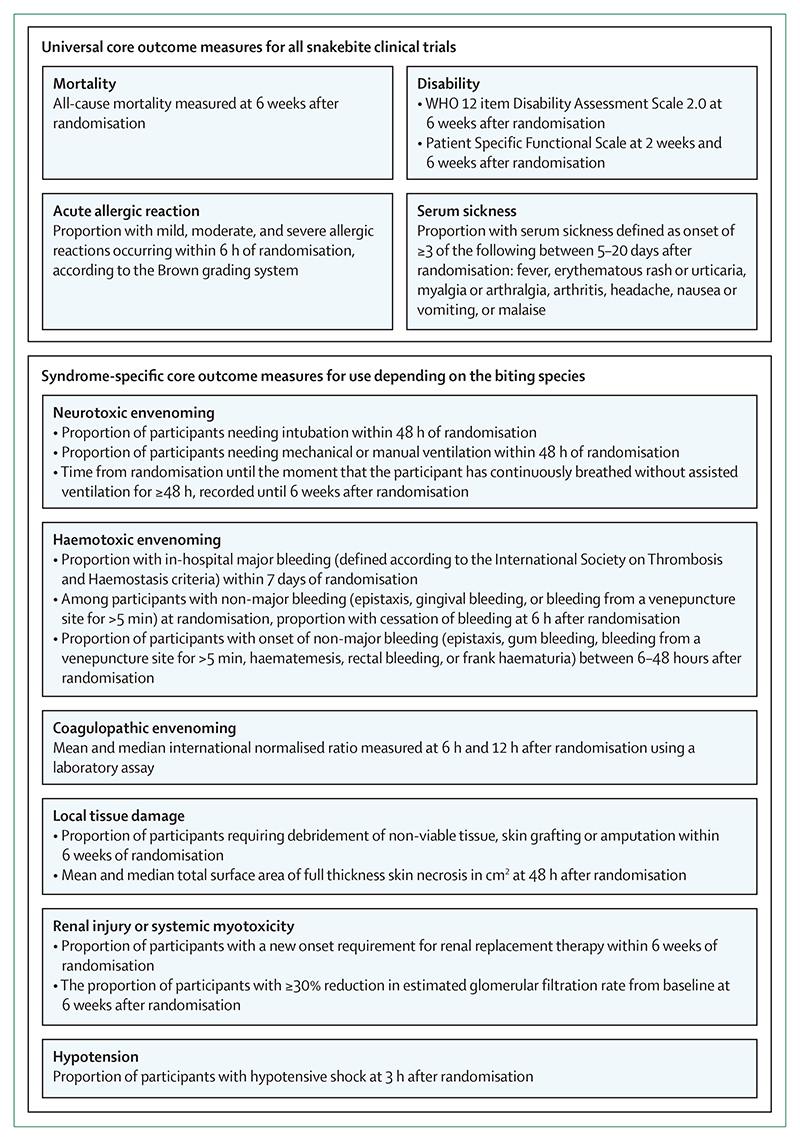
Overview of the core outcome measurement set The full core outcome measurement set, including definitions, is available (appendix p 48).
