# Supplementary material for: A global core outcome measurement set for snakebite clinical trials
Source: Lancet Glob Health. Author manuscript; Available in PMC 2025 May 16. (PMC7617674; doi:10.1016/S2214-109X(22)00479-X)
Supplement: Supplementary appendix [file EMS202197-supplement-Supplementary_appendix.pdf]

THE LANCET
Global Health

Supplementary appendix

This appendix formed part of the original submission and has been peer reviewed. We post it as supplied by the authors.

Supplement to: Abouyannis M, Esmail H, Hamaluba M, et al. A global core outcome measurement set for snakebite clinical trials. *Lancet Glob Health* 2023; **11:** e296–3300.

Supplementary Appendix

A global core outcome measurement set for snakebite clinical trials

Michael Abouyannis, MBChB[1,2*], Hanif Esmail, PhD[3,4], Mainga Hamaluba, MD[2,5], Mwanajuma Ngama, MSc[2], Hope Mwangudzah, BSc[2], Noni Mumba, MA[2], Betty K. Yeri, BSc[2], Salim Mwalukore MA[2], Hassan J. Alphan BSc[2], Dinesh Aggarwal, MBBS[6], Gabriel Alcoba, MD[7,8], Nick Cammack, PhD[9], Jean-Philippe Chippaux, MD[10], Matthew E. Coldiron, MD[11], José M. Gutiérrez, PhD[12], Abdulrazaq G. Habib, FAS[13], Robert A. Harrison, PhD[1], Geoffrey K. Isbister, MD[14], Eric J. Lavonas, MD[15], Diogo Martins, MD[9], Isabela Ribeiro MD[16], James A. Watson, DPhil[5,17], David J. Williams, PhD[18], Nicholas R. Casewell, PhD[1], A. Sarah Walker, PhD[3], and David G. Lalloo, FRCP[1], on behalf of the Snakebite Global Core Outcome Set Study Group

[1] Centre for Snakebite Research and Interventions. Liverpool School of Tropical Medicine, Liverpool, UK
[2] KEMRI-Wellcome Research Programme, Kilifi, Kenya
[3] MRC clinical trials unit at UCL, London, UK
[4] Institute for Global Health, University College London, London, UK
[5] Centre for Tropical Medicine & Global Health, Nuffield Department of Medicine, Oxford, UK
[6] Department of Medicine, University of Cambridge, UK
[7] Medical dpt., Médecins Sans Frontières/Doctors Without Borders, Geneva, Switzerland
[8] Division of Tropical and Humanitarian Medicine, Geneva University Hospitals, Geneva, Switzerland
[9] Wellcome Trust
[10] University of Paris Cité, IRD, MERIT, F-75006 Paris, France
[11] Epicentre, Paris, France
[12] Instituto Clodomiro Picado, Facultad de Microbiología, Universidad de Costa Rica, San José 11501, Costa Rica
[13] Bayero University, Kano, Nigeria
[14] Clinical Toxicology Research Group, University of Newcastle, Newcastle, New South Wales, Australia
[15] Department of Emergency Medicine, Denver Health and Hospital Authority, Denver, Colorado, USA; Department of Emergency Medicine, University of Colorado School of Medicine, Aurora, Colorado, USA
[16] DNDi - Drugs for Neglected Diseases initiative, Geneva, Switzerland
[17] Mahidol Oxford Research Unit, Faculty of Tropical Medicine, Mahidol University, Bangkok, Thailand
[18] Regulation and Prequalification Department, Access to Medicines and Health Products Division, World Health Organization, Geneva, Switzerland

* Corresponding author:
Email: michael.abouyannis@lstmed.ac.uk (MA)

Table of Contents

A GLOBAL CORE OUTCOME MEASUREMENT SET FOR SNAKEBITE CLINICAL TRIALS 1

MEMBERS OF THE SNAKEBITE GLOBAL CORE OUTCOME SET STUDY GROUP (EXPERT GROUP) 3

RELEVANT AFFILIATIONS OF THE STAKEHOLDER GROUP MEMBERS 4

THE SHORTLISTING QUESTIONNAIRE 5

ADDITIONAL OUTCOME MEASURES RECOMMENDED BY THE EXPERT GROUP AND STAKEHOLDER GROUP 13

THE INFORMATION PACK 14

- Using this document 15
- Mortality outcome measures 16
- Neurotoxicity outcome measures 17
- Coagulopathy outcome measures 19
- Haemorrhage outcome measures 21
- Renal injury outcome measures 23
- Local tissue damage outcome measures 26
- Systemic myotoxicity outcome measures 28
- Disability scales 29
- Adverse event outcome measures 36
- Information Pack References 38

OVERVIEW OF THE SHORTLISTING QUESTIONNAIRE SCORES AND THE CONSENSUS MEETING VOTE RESULTS ON POTENTIAL CORE OUTCOME MEASURES 41

OUTCOME MEASUREMENT INSTRUMENT REFERENCES 46

THE FULL CORE OUTCOME MEASUREMENT SET 48

- Mortality: 48
  - *All-cause mortality* ☏ *48*
- Disability scales: 48
  - *WHODAS 2.0* ☏ *48*
  - *Patient-specific functional scale* ☏ *48*
- Adverse events: 48
  - *Brown grading of allergic reactions* *48*
  - *ASP defined serum sickness* ☏ *48*
- Neurotoxic envenoming: 49
  - *Need for intubation and ventilation* *49*
- Haemotoxic or coagulopathic envenoming: 50
  - *ISTH defined major bleeding* *50*
  - *Clinically relevant non-major bleeding (CRNMB)* *50*
  - *INR* *50*
- Local tissue damage: 50
  - *Requirement for surgery* ☏ *50*
  - *Total surface area of skin necrosis* *51*
- Renal injury or systemic myotoxicity: 51
  - *Requirement for renal replacement therapy* *51*
  - *≥30% reduction in eGFR from baseline at 42 days* *51*
- Hypotension: 51
  - *Hypotensive shock at 3-hours* *51*
- Overview of Core Outcome Measure Timepoints 52

THE CORE OUTCOME MEASUREMENT SET OUTCOME MEASUREMENT INSTRUMENTS AND DEFINITIONS 53

- 12-item WHODAS 2.0 Outcome Measurement Instrument 53
- 12-item WHODAS-child Outcome Measurement Instrument 54
- Patient Specific Functional Scale Outcome Measurement Instrument 55
- Brown Grading of Allergic Reactions Outcome Measurement Instrument 56
- Age Specific Definitions of Hypotensive Shock in Children 56
- Australian Snakebite Project (ASP) definition of serum sickness 56
- International Society on Thrombosis and Haemostasis (ISTH) Definition of Major Bleeding 56

Members of the Snakebite Global Core Outcome Set Study Group (Expert Group)

Dr Chanaveerappa Bammigatti, MD
Dr Rebecca W Carter, PhD
Professor Charles John Gerardo, MD
Professor H. Janaka de Silva, DPhil
Dr Thomas Lamb, MBChB
Dr Matthew R Lewin, PhD
Dr Wuelton Monteiro, PhD
Professor Ymkje Stienstra, MD

Relevant Affiliations of the Stakeholder Group Members

The African Snakebite Research Group, Drugs for Neglected Diseases initiative, French National Research Institute for Sustainable Development, Global Snakebite Initiative, Instituto Clodomiro Picado, KEMRI-Wellcome Trust Research Programme, Liverpool School of Tropical Medicine (LSTM) Centre for Snakebite Research and Interventions, the Mahidol Oxford Tropical Medicine Research Unit, Médecins Sans Frontières, the Medical Research Council Clinical Trials Unit at University College London, the Wellcome Trust, and the World Health Organization.

The Shortlisting Questionnaire

**Study title: a globally relevant core outcome set for snakebite trials**	
**This document: outcome measure shortlisting questionnaire**	

Guide to completing this questionnaire:

-Refer to the 'information pack' whilst completing this questionnaire
-All summary data from this questionnaire will be anonymised.
-Aim to complete all sections: even if you feel you lack experience in a certain area.
-In section 3, outcome domains from the systematic review are listed (as well as additional outcome measures which have been suggested by the stakeholder group). Mark each outcome as 'essential', 'desirable' or 'inappropriate' and score from 1 to 9. This judgement should be based on your overall view of each outcome's validity, reliability, acceptability and feasibility. Provide brief justification for each item marked as 'inappropriate' (ideally one short sentence). There is no need to justify inclusions (essential/desirable). If after referring to the information pack you feel you are not able to make an informed judgement of the outcome measure, you can select 'don't know'.
-Section 4: list any outcome measures that you think might be useful for a core outcome set, that have not been captured in section 3. There is no limit to this section. Ensure suggested outcome measures are valid, reliable, acceptable and feasible, and provide concise but adequate detail to clearly define what is being measured.
-Section 5: list any information or data that you feel is appropriate for inclusion in the information pack

-The scope of this core outcome set will be to provide a list of outcome measures for use in all phase II and III clinical trials whether based in low or high income settings. Outcomes related to mortality, disability assessment and adverse events will be applied universally to all trials. The use of outcome measures related to specific syndromes of envenoming (such as neurotoxicity or coagulopathy) will be adaptable so that trialist can select outcome measures that are relevant to the syndrome of envenoming which is being studied (i.e., neurotoxic outcome measures for non-spitting cobras and coagulopathic/haemorrhagic and local tissue damage outcome measures for *Echis ocellatus.*
-For each outcome measure, consider whether it is appropriate for a trial recruiting participants with a bite by a snake species causing that pathology (i.e., for renal injury based outcome measures, picture a trial on antivenom for treating Russell's viper)

Section 1. Background information	Respond below:
Name:	
Institution:	
Role:	

Are you a clinician (Yes/No):<br>Are you a clinician with experience of treating snakebite (Yes/No):<br>Are you an academic with an interest in snakebite (Yes/No):<br>Have you led or co-investigated a clinical trial in any disease area (Yes/No):<br>Have you led or co-investigated a clinical trial in snakebite (Yes/No):	

Section 2. Selection of outcome categories

For the purposes of developing a globally relevant core outcome set, we plan to categorise outcome measures as follows:
-Mortality
-Neurotoxicity
-Coagulopathy
-Haemorrhage
-Renal injury
-Local tissue damage
-Systemic myotoxicity
-Patient-centred outcomes/disability scales
-Adverse events

	**Respond below:**
1) Are these categories appropriate? ('Yes'/'No')	
2) If not appropriate, briefly state why? (Free text)	

Section 3. Shortlisting outcome measures

This section lists all the outcome domains that were extracted from snakebite clinical trials during the systematic review. Over 150 outcome measures were extracted, so these have been grouped into domains to streamline shortlisting. There may be different ways to measure each outcome, such as different time points, definitions, or specific assays. These details will be considered at the next stage of COS development. For each outcome, selects whether it is essential, desirable, or inappropriate for use in a COS. If it is inappropriate, provide 1-2 brief sentences to explain why. Consider the outcome's clinical significance, importance to patients, feasibility, reliability, and acceptability.

Domain	Score from 9-1 (9-7 = essential outcome measure; 6-4 = desirable outcome	If outcome is inappropriate for use in a core outcome set, provide 1-2 sentences to explain why

	**measure; 3-1 = inappropriate outcome measure)**	
Example 1: Bilirubin	*1 - inappropriate*	*Bilirubin not associated with envenoming. Not a valid outcome.*
Example 2: mortality	*9 - essential*	*-*
Example 3: Tongue fasciculations	*1 - inappropriate*	*Subtle clinical sign that would not be reliably detected*
**Mortality**		
All-cause mortality		
Cause specific mortality		
In-hospital mortality only		
In-hospital mortality with community follow-up for snakebite associated deaths following discharge (for example: 'deaths occurring at 30 days following randomisation')		
**Neurotoxicity**		
Requirement for invasive ventilation		
Duration of invasive ventilation		
Bulbar palsy		
Ptosis		
Extraocular muscle palsy		
Assessment of functional ability (such as the GBS score at 4 weeks - see information pack p7)		
Spirometry (such as forced vital capacity)		
Electromyography		
Measures of non-facial muscle weakness (such as hand grip strength)		
**Coagulopathy**		
20-minute whole blood clotting test		
Lee White clotting test		
Prothrombin time		

INR - lab based	
Bedside INR meter (handheld device)	
aPTT	
Fibrinogen quantification	
Platelet count	
Clotting factor quantification (such as factors V or X)	
Thromboelastography (TEG) or Thromboelastometry (ROTEM)	
Fibrin and Fibrinogen-Degradation Products quantification (including D-dimer)	
Clotting factor transfusion requirement	
**Haemorrhage**	
Time to cessation of local or systemic bleeding - of bleeding events not reaching the threshold for the ISTH criteria	
ISTH defined major bleeding (see information pack p10)	
EMA defined clinically relevant non-major bleeding (see information pack p10)	
Haemoglobin to quantify anaemia	
Bleeding index (see information pack p11)	
Requirement for whole blood or red blood cell transfusion	
Reticulocyte count	
**Renal injury**	
Acute kidney injury KDIGO criteria (see information pack p 12)	
Acute kidney disease (such as a 30% decline in eGFR from baseline at 30 days after randomisation) (see information pack 12-14)	
CKD (at ≥3 months after randomisation and staged according to KDIGO eGFR criteria) (see information pack p13)	
Requirement for renal replacement therapy	
MAKE (major adverse kidney events) composite endpoint (see information pack p14)	

Existing renal core outcome set for peri-operative trials (see information pack p14)	
Serum urea	
Proteinuria quantification (such as albumin:creatinine ratio)	
Proteinuria detection using urine dip	
Microscopic haematuria on urine dip	
**Local tissue damage**	
Swelling measured by circumference of bitten limb	
Swelling measured as length of proximal extension	
Swelling measured as a binary outcome of greater or less than half of the length of the bitten limb	
Swelling measured by limb volume (e.g., displacement of water)	
Number developing skin blisters	
Total surface area of skin blisters	
Number with skin necrosis	
Total surface area of skin necrosis - measured with tape measure<br>Total surface area of skin necrosis - measured with digital technology (photograph and software to estimate surface area) (see information pack p15)	
Need for amputation, skin grafting or debridement	
Pain - measured using an ordinal scale	
Opioid requirement	
Numbers developing skin and soft tissue infection	
Serum creatine kinase (as a measure of severity of local myonecrosis)	
The use of a disability scale as an outcome measure local tissue damage	
**Systemic myotoxicity**	
Serum creatinine kinase - peak level	

Serum creatine kinase - area under the curve	
Measures of renal injury (either AKI, AKD or CKD) as an endpoint for rhabdomyolysis	
Myoglobinuria on urine dip	
Myalgia	
**Disability scales**	
Disabilities of the arm, shoulder, and hand score (DASH score) (see information pack p18)	
Lower extremity functional scale (LEMS) (see information pack p19)	
Patient-reported outcome measurement information system physical function-10 score (PROMIS PF-10) (see information pack p20)	
Patient-specific functional scale (PSFS) (see information pack p20-21)	
Patient's global impression of change-1 instrument (see information pack p21)	
The physical function domain of the SF-36 questionnaire (see information pack p21)	
World Health Organization Disability Assessment Schedule (WHODAS) (see information pack p21-22)	
**Adverse events**	
NIAID-FAAN defined anaphylaxis (see information pack p23)	
Brown grading system for anaphylaxis (see information pack p23)	
ASP study definition of serum sickness (see information pack p24)	
The below outcomes were extracted during the systematic review but have not been included in the information pack:	
**Cardiotoxicity**	
Hypotension	
**Venom antigenaemia**	
Venom antigen quantification	
**Additional antivenom requirement**	
Total dose of antivenom required	

Need for additional antivenom following initial dosing	
**Functional status**	
Measure of limb weakness (such as MRC scale of strength)	
Number of physiotherapy or occupational therapy sessions attended	
Time to return to work	
**Others**	
Duration of hospital admission	
Anosmia	
GI symptoms of envenoming	
Hypoxic brain injury	
Leucocyte count	
Serum lactate dehydrogenase	
Urine neutrophil gelatinase associated lipocalin (NGAL)	
Serum metalloproteinase	

Section 4

List any outcome measures that you think might be useful for a core outcome set and are not captured in section 2.
There is no limit to this section. Ensure suggested outcome measures are valid, reliable, feasible. Provide adequate detail to clearly define what is being measured.

**Example:**	*Serial measurement of serum lactate as a marker of tissue perfusion*
**1**	
**2**	
**3**	
**4**	
**5**	
**6**	
**7**	

**8**	
**9**	
**10**	
**Section 5**	
List any additional evidence that has not been included in the information pack that you feel is important for guiding decisions on an appropriate core outcome set (for example, data highlighting that a surrogate marker is associated with mortality or disability)?	

Additional Outcome Measures Recommended by the Expert Group and Stakeholder Group

Additional outcome domains proposed by the stakeholder group and expert group:

1. Assessment of functional ability (such as the Guillain-Barré syndrome scale) as a measure of recovery from neurotoxicity.
2. INR measured using a point of care device.
3. Thromboelastography.
4. European Medicines Agency defined clinically relevant non-major bleeding.
5. Bleeding index (calculated by measuring change in haemoglobin and adjusting for quantity of blood transfused in unit).
6. Acute kidney disease.
7. Chronic kidney disease.
8. Major adverse kidney events (MAKE) composite endpoint.
9. An existing renal core outcome set for peri-operative trials.
10. Proteinuria quantification (such as albumin:creatinine ratio).
11. Proteinuria measured by urine dip.
12. Urine neutrophil gelatinase associated lipocalin.
13. Surface area of skin necrosis measured using digital technology.
14. Use of a disability scale as a measure of local tissue damage.
15. Measures of renal injury as an outcome for rhabdomyolysis.
16. World Health Organization Disability Assessment Schedule.
17. Australian Snakebite Project study definition of serum sickness.

Information pack – overview of potential outcome measures for snakebite clinical trials

Study title: A globally relevant core outcome set for snakebite trials

Using this document

This is a working document and your input into updating and improving the information herein is encouraged. The aim of this document is to support informed decisions on optimum outcome measures, and to ensure we are basing our decisions on the available evidence.

The core outcome set is planned to be adaptive with nine categories of outcome measures, as follows: mortality, neurotoxicity, coagulopathy, haemorrhage, renal injury, local tissue damage, systemic myotoxicity, disability scales and adverse events. Three categories are anticipated to be relevant to all snakebite trials: mortality, disability scale(s) and adverse events. The other categories will be selected by trialists, depending on the population being studied. For example, a trial in West Africa on *Echis ocellatus* envenoming would select the following categories: mortality, coagulopathy, haemorrhage, local tissue damage, disability scale(s), and adverse events. Platform trials can select outcome measure categories for different groups of participants. For example, a multi-centre platform trial could use the neurotoxicity category outcome measures for participants with clinical signs of neurotoxic envenoming, and coagulopathy category outcome measures for those participants with a coagulopathy.

The outcomes within each category will represent the minimum outcome measures that should be used in all future human clinical efficacy trials (Phase II and III) in any region including high and low-income settings. Trialists will not be expected to use these as primary outcome measures, and they can use any other outcome measures they wish. By having a core set of outcome measures, quality of outcome reporting can be improved, and data can be compared and combined, including in meta-analyses.

This document aims to provide a concise summary of the key features of various potential outcome measures that could be included in a core outcome set. These outcome measures have been identified through a systematic review of all randomised controlled trials of snakebite. Where relevant, additional outcome measures have been identified from the literature.

Outcome measures should be assessed in terms of reliability, validity, feasibility (cost and equipment requirements), acceptability (to participants) and clinical significance. All of the outcome measures must be relevant to both low-income and high-income settings. When considering feasibility, it is important regard the context as an appropriately funded clinical trial. Whether or not an assay is normally available in a low-income rural health facility does not exclude its use, as long as it can feasibly be used in a good quality clinical trial in this setting. Where possible, outcome measures of high clinical significance should be included. To support smaller clinical trials, surrogate endpoints may be included. The use of surrogate endpoints, or endpoints of low clinical significance, must be justified, including by validity data demonstrating an association with clinically significant endpoints.

You will receive a study questionnaire, which you can use to suggest any additional outcome measures that have not been captured in the systematic review. Any suggestions for relevant information or recommendations of relevant studies that should be included in this document are welcome and can be emailed to: michael.abouyannis@lstmed.ac.uk. The information included in this document is likely to influence the group’s choices on core outcomes, and it is therefore important that the information is representative of the available literature.

Mortality outcome measures

Summary results from systematic review of snakebite RCTs and protocols of ongoing RCTs		
**Outcome measure**	**Number of unique methods for recording the outcome measure**	**Number of studies using the outcome measure**
Mortality	1	18

Mortality is usually included as an outcome measure in core outcome sets. It is important to consider whether all-cause mortality is used, the duration of follow-up and whether participants are censored on hospital discharge.

All-cause mortality

The advantage of measuring all-cause mortality is that it is simple and that it includes deaths that may be indirectly due to snakebite. Identifying specific causes of death is often unreliable and can introduce bias.

Duration of follow-up and whether to censor on hospital discharge

In the majority of snakebite trials conducted to date, participants have been censored on hospital discharge and the duration of follow-up has been dependent on the duration of the hospital admission. This approach is convenient for trialists and participants and reduces costs. However, deaths following discharge may be missed. This is particularly true in rural LMIC settings where there may be barriers to reaching healthcare, and systems for reporting deaths may be less reliable.

There are cost implications to arranging face to face follow-up visits, such as participant remuneration. Now that mobile phones are widely available, the majority of participants could be followed-up by telephone.

Certain cases may have a prolonged hospital admission prior to their death. Such as participants that are intubated or receiving renal replacement therapy. It would be important to use a follow-up period that would capture such deaths.

Neurotoxicity outcome measures

Summary results from systematic review of snakebite RCTs and protocols of ongoing RCTs		
**Outcome measure**	**Number of unique methods for recording the outcome measure**	**Number of studies using the outcome measure**
Ptosis	3	6
Requirement for invasive ventilation	2	6
Duration of invasive ventilation	3	4
Extraocular muscle palsy	2	3
Measure of other skeletal muscle weakness	2	3
Bulbar palsy	3	2
Electromyography	1	2
Spirometry	3	1

Need for intubation and ventilation

Neurotoxic envenoming can lead to primary ventilatory failure. Emergency treatment is supportive and includes intubation and mechanical ventilation. The decision of when to intubate is complex and is based on the assessment of multiple objective and subjective parameters, which cannot be pre-specified based on specific criteria.[2,3] Of 14 snakebite clinical trials of neurotoxic envenoming, six recorded the number of participants requiring intubation and ventilation.

Although this outcome measure is clinically important, a limitation is that variations in availability of intensive care beds will bias comparisons between trials conducted in different geographic regions. The populations of most rural LMIC regions do not have access to intubation and ventilation.

Duration of intubation and ventilation

Of 14 snakebite clinical trials of neurotoxic envenoming, four recorded the duration of invasive ventilation. None of these trials provided a clear definition for the duration of ventilation. The start point can be defined as the time of randomisation or the time of intubation. The endpoint can be defined as: the 1st extubation; successful extubation; successful spontaneous breathing trial; or successful weaning.[4] Recording re-intubation, and the use of non-invasive ventilation during weaning, adds further complexity to this outcome measure.

In a recently published core outcome set for ventilation trials, duration of ventilation was defined as the time between randomisation and first successful unassisted breathing (or death).[1] The term 'unassisted breathing' was defined as "being free from invasive ventilation, including extracorporeal lung support and non-invasive ventilation delivering volume or pressure support."[1] Administration of continuous positive airway pressure and high-flow oxygen therapy were not classified as forms of assisted breathing, as these devices predominantly modify oxygen delivery, rather than provide ventilatory support. 'Success' was defined as not requiring assisted ventilation for a 48-hour period.

Other ventilation outcome measures that were included in the previously published core outcome set were: date and time of extubation; date and time of establishing a natural airway; and date and time of each reintubation.

Assessment of bulbar palsy

Bulbar palsy describes an impairment of the lower cranial nerves (VII-XII). This can cause difficulty in swallowing and speaking. Bulbar palsy in snake envenoming is a concerning sign as it may precede aspiration or choking.

Standardising the assessment of bulbar palsy may be challenging. Tools for grading the severity of bulbar palsy are available, but these have been developed for chronic conditions such as motor neuron disease and are unlikely to be useful for snakebite.[5]

Ptosis and Extra-ocular muscle palsy

Extraocular muscle palsy is identified on the clinical examination of eye movements. The earliest sign is often of diplopia (double vision). Ptosis describes the presence of upper eyelid weakness. Extraocular muscle palsy has been used as an outcome measure in three snakebite clinical trials, with a combined sample size of 108. Ptosis has been measured in six snakebite trials with a combined sample size of 178. Detection of these clinical sign suggest systemic neurotoxic envenoming, indicate the need for antivenom, and may precede further deterioration such as ventilatory failure. However, these signs are of less clinical significance than bulbar palsy, and not all patients with ptosis or ophthalmoplegia will require intubation. Some trials have attempted to severity grade ptosis, based on the amount of iris that is obscured. Such assessments have not been validated.

Assessment of functional ability

Snakebite trials have rarely provided an assessment of functional weakness, such as ability to walk. There is no validated tool for assessing physical function following neurotoxic envenoming. In Guillain-Barré syndrome (GBS), a disease with similar clinical features, the 'GBS disability score' at 4 weeks after randomisation is the established primary outcome for clinical trials.[6] This score is simple and assesses parameters of clinical significance. This tool could be adapted for use in snakebite trials.

**GBS disability score**	
1.	Healthy
2.	Minor symptoms or signs of neuropathy but capable of manual work
3.	Able to walk without support of a stick but incapable of manual work
4.	Able to walk with a stick, appliance or support
5.	Confined to bed or chair bound
6.	Requiring assisted ventilation
7.	Dead

Spirometry

One snakebite clinical trial published by Watt et al in 1986, with a sample size of 10 participants, included measurement of spirometry as an outcome measure. The parameters that were measured included forced vital capacity, maximal pressure on blowing into the tubing of a sphygmomanometer, and negative inspiratory pressure. Forced vital capacity is the most established of these measures. Vital capacity is the maximum volume of air an individual can expel following a maximum inspiration. It should be noted that measurement of FVC has not been adopted in clinical trials in other diseases causing acute muscle paralysis. Amongst 12 trials included in a systematic review of Guillain-Barré syndrome, only one assessed vital capacity as an outcome measure.[7]

Electromyography

Two snakebite trials, with a combined sample size of 60 participants, have measured electromyography. Electromyography is not widely available, requires specialist equipment and specialist training. It may be less acceptable to patients as it is invasive. Although electromyography may be useful in certain trials, such as a phase II proof of concept study, it would be challenging to deliver to all participants in a phase III clinical trial.

Coagulopathy outcome measures

**Summary results from systematic review of snakebite RCTs and protocols of ongoing RCTs**		
**Outcome measure**	**Number of unique methods for recording the outcome measure**	**Number of studies using the outcome measure**
Bedside clotting tests	18	30
- 20-minute whole blood clotting test	(9)	(15)
- Lee White clotting time	(6)	(12)
- Bleeding time	(1)	(2)
- Other bedside clotting assay	(2)	(3)
Fibrinogen quantification	5	17
Clotting studies (INR, PT, APTT)	9	14
Platelet count	3	9
Clotting factor quantification	6	7
Fibrin and Fibrinogen-Degradation Products quantification	5	6
Clotting factor replacement	1	2

Introduction to venom induced consumption coagulopathy (VICC)

Venom induced consumption coagulopathy (VICC) is caused by a wide variety of snake species including vipers, Australasian elapids and certain colubrids.[8] It is triggered by the procoagulant activity of various venom components.[8]

The procoagulant venoms components act late in the coagulation pathway, by activating prothrombin or factor X. As this is part of the common pathway, prolongation of both the activated partial thromboplastin time (aPTT) and the prothrombin time (PT- which is equivalent to INR) occur. As fibrinogen is degraded, serum fibrinogen levels fall, and fibrinogen degradation products rise.

Bedside clotting tests

Measures of bedside clotting were used in 30 of the 58 studies identified in our systematic review of snakebite trials. The 20-minute whole blood clotting test (20WBCT) was used in 15 studies from various regions including Africa and Asia. This test involves adding 2mL of whole blood to a glass tube and leaving it undisturbed for 20 minutes. The tube is then inverted and if the blood has not clotted the result is abnormal.

This test is cheap and simple to undertake in rural LMIC settings. Its accuracy to predict coagulopathy compared to laboratory-based assays, such as fibrinogen quantification or prothrombin time, has shown sensitivity of approximately 80% and specificity of approximately 95%, although results have varied significantly between studies. A disadvantage of the 20WBCT is that it is a binary outcome, whereas the established laboratory assays are continuous measures.

The Lee White clotting time (LWCT) is similar to the 20WBCT, except that the tube is undisturbed for 5 minutes, then repeatedly inverted each minute until a clot has formed. The threshold for defining an abnormal result has varied in snakebite clinical trials, ranging from 10 to 16 minutes.[9,10] The LWCT was used exclusively in clinical trials based in Asia and South America. In an observational study of *Bothrops* spp envenoming, the LWCT was found to be 78% sensitive and 41% specific to detect hypofibrinogenaemia.[11]

Other bedside clotting tests, such as ‘bleeding time’ or ‘clot quality’ have scarcely been used and are not validated.

Clotting studies (PT, INR and aPTT)

Both PT and aPTT are raised in VICC. The INR is derived by comparing the PT to a control PT, which is based on a standardised thromboplastin reagent.

Clotting studies have been used in 14 of the snakebite trials identified in the systematic review (9 measured INR; 8 measured PT; and 3 measured aPTT). Cut-offs for INR included <1, <1.3 and <2. Time point of measurement of clotting studies was often 6 hours, although many studies reported the time taken for clotting studies to fall below a certain threshold.

It is difficult to delineate the PT/INR and aPTT in terms of which is more accurate for detecting VICC. An evaluation using data from the Australian Snakebite Project recommended that both PT and aPTT be used as laboratory end points.[12] The aPTT was found to normalise more rapidly than the PT (median time of 5 hours vs 9 hours), but this data is based on Elapid envenoming, whereas Viperids are the commonest cause of coagulopathy in other regions.

The advent of bedside INR meters offers a convenient solution to measuring coagulopathy in rural LMIC settings, although these have not been validated for use in snake envenoming.

Fibrinogen concentration

Fibrinogen levels fall due to consumption in VICC. It is challenging to assess whether measuring fibrinogen concentration offers any advantages over standard measures of clotting, such as PT, INR and aPTT. Data from the Australian Snakebite Project found that the majority of patients with abnormal fibrinogen have abnormal clotting studies, and suggests that measuring fibrinogen adds little value.[12] The fibrinogen assay is less widely available than clotting studies, even in resource rich settings such as Australia. Fibrinogen concentration directly measures clotting factor consumption, whereas clotting studies provide a meaningful measure of clotting function.

Haemorrhage outcome measures

Summary results from systematic review of snakebite RCTs and protocols of ongoing RCTs		
**Outcome measure**	**Number of unique methods for recording the outcome measure**	**Number of studies using the outcome measure**
Cessation of local or systemic bleeding	4	9
Anaemia	3	4
ISTH defined major bleeding	1	4
Blood transfusion requirement	1	3

Cessation of local or systemic bleeding (not including major bleeding)

These outcome measures have often been poorly defined in snakebite trials. Common bleeding points include the gums, venepuncture sites and the bite site. Low volume blood loss at these sites is of limited clinical importance, although it does suggest systemic envenoming and the need for antivenom. In this sense, minor bleeding events are a surrogate marker of systemic envenoming. Measures of coagulopathy offer an alternative surrogate marker that is more sensitive and can be quantified. The additional value of using outcome measures based on non-clinically significant bleeding events needs to be considered.

Definitions of major bleeding

Four clinical trials of snakebite have used the ISTH (International Society on Thrombosis and Haemostasis) definition. The ISTH definition was developed for use as a safety outcome for clinical trials of anticoagulant therapies and is widely accepted in this field. It identifies bleeds that are life threatening, cause chronic impairment or require major healthcare resources to manage.[13]

ISTH defined major bleeding	
1.	Fatal bleeding OR
2.	Symptomatic bleeding in a critical area or organ (such as intracranial, intraspinal, intraocular, retroperitoneal, intraarticular or pericardial, or intramuscular with compartment syndrome) OR
3.	Bleeding causing a fall in hemoglobin level of 2 g/dL (1.24 mmol/L) or more, or leading to transfusion of two or more units of whole blood or red cells.

Alternatives to the ISTH criteria include the TIMI, Gusto and ISCOAT bleeding classifications. These have not been considered further as they are less specific for clinically meaningful bleeding and are less well established.

The European Medicines Agency (EMA) accepts the ISTH definition, with the addition of one parameter: ‘bleeding that necessitates surgical intervention.’

A definition of clinically relevant non-major bleeding is also available from the EMA. It should be highlighted that this was developed as a safety outcome for clinical trials of anticoagulants and may not be valid for snakebite associated haemorrhage.

EMA clinically relevant non-major bleeding*
Multiple source bleeding
Spontaneous haematoma >25cm^2
Traumatic haematoma >100cm^2
Intramuscular haematoma on ultrasound and absence of compartment syndrome
Excessive wound haematoma
Macroscopic haematuria
Epistaxis or gingival bleeding requiring tamponade or other intervention
Bleeding after venipuncture for >5 minutes
Haemoptysis, haematemesis or rectal bleeding requiring endoscopy or other intervention

*There are additional criteria that are specific for anticoagulation. Such as bleeding that requires the discontinuation of anticoagulation therapy.

Anaemia

Outcome measures based on packed cell volume or haematocrit have been used in four snakebite clinical trials. All of these trials reported the fall in haematocrit or PCV (mean fall, peak fall or fall between admission and discharge). Both haemoglobin concentration and haematocrit are likely to be similarly accurate for detecting anaemia in participants with acute blood loss.[14] Haemoglobin is more widely accepted for diagnosing anaemia and is used in the ISTH criteria for major haemorrhage.

There are limitations of using haemoglobin concentration as an outcome measure. During acute blood loss, the haemoglobin will not fall until plasma volume has been restored and dilution has taken place. Dehydration and venom-induced capillary leak can result in under estimation of blood loss. Therefore, assessments of anaemia should be delayed at least 48 hours after a bleeding event and once the participant is fluid replete.

Severely anaemic participants or those with severe active bleeding may be offered blood transfusion which will raise the haemoglobin and would require adjustment. The EMA recommend calculating the bleeding index:[15]

Bleeding index = (Baseline haemoglobin) - (end of treatment haemoglobin) + (the number of units of blood transfused)

Blood transfusion requirement

This can be calculated as the total number of units of packed red cells or whole blood transfused during the trial follow-up period. There may be differences between study sites based on background levels of anaemia, the haemoglobin threshold for offering blood transfusion, and the availability of blood products.

Renal injury outcome measures

Summary results from systematic review of snakebite RCTs and protocols of ongoing RCTs		
**Outcome measure**	**Number of unique methods for recording the outcome measure**	**Number of studies using the outcome measure**
Acute kidney injury (non-specific criteria)	4	6
Requirement for renal replacement therapy	1	6
Serum creatinine	2	5
Serum urea	1	3
Acute kidney injury (RIFLE or KDIGO criteria)	1	2
Haematuria	1	1

Acute kidney injury

There are three internationally recognised criteria for defining acute kidney injury (AKI). Two of these have been used in snakebite clinical trials ('RIFLE' and 'KDIGO'). The KDIGO criteria are the most recently developed criteria and replace RIFLE and AKIN. One advantage of the KDIGO criteria is that it does not require a baseline creatinine to diagnose AKI. A further advantage is that the KDIGO criteria provide a definition of AKI for children and neonates.[16,17] A validation study in ICU patients in Brazil found that the RIFLE, AKIN and KDIGO criteria were similarly accurate in predicting mortality.[18]

**KDIGO definition of AKI**[17]
Increase in serum creatinine by ≥0.3 mg/dl (≥26.5 µmol/l) within 48 hours; OR
Increase in serum creatinine to ≥1.5 times baseline, which is known or presumed to have occurred within the prior 7 days; OR
Urine volume <0.5 ml/kg/h for 6 hours.

**KDIGO staging of AKI**[17]		
**Stage**	**Serum creatinine**	**Urine output**
1	1.5–1.9 times baseline<br>OR<br>≥0.3 mg/dl (≥26.5 mmol/l) increase	<0.5 ml/kg/h for 6–12 hours
2	2.0-2.9 times baseline	<0.5 ml/kg/h for ≥12 hours
3	3.0 times baseline<br>OR<br>Increase in serum creatinine to ≥4.0mg/dl (≥353.6 mmol/l)<br>OR<br>Initiation of renal replacement therapy OR, in patients <18 years, decrease in eGFR to <35 ml/min per 1.73 m2	<0.3 ml/kg/h for ≥24 hours<br>OR<br>Anuria for ≥12 hours

It should be considered that short term changes in serum creatinine and urine output are not necessarily clinically significant. The FDA does not recognise a diagnosis of AKI as an outcome measure in drug registration trials.[19] Although staging of AKI has been validated in multiple studies and does correlate with mortality, an irreversible loss of kidney function after AKI represents a more clinically significant outcome measure.

Acute kidney disease

Acute kidney disease (AKD) is defined as the persistence of the KDIGO criteria of AKI stage ≥1, for a period of 7-90 days. After 90 days, this would be defined as chronic kidney disease (CKD). A staging system for AKD has been proposed, although various definitions exist.

**Proposed staging system for AKD**[20]

Stage	Criteria
1	Serum creatinine 1.5x baseline
2	Serum creatinine 2x baseline
3	Serum creatinine 3x baseline or renal replacement therapy

There is less consensus on the definition or staging of AKD, compared to AKI and CKD. For example, the KDIGO guidelines identify AKD as an umbrella term for any acute structural or functional kidney abnormality, and do not recognise the above staging system for AKD.[17] A previously published core outcome set for renal endpoint in peri-operative trials (detailed below) defined AKD as: ≥30% decline in estimated glomerular filtration rate (eGFR) from baseline at 30 days after operation in a patient who previously met creatinine-based KDIGO criteria for AKI within 7 days of surgery

Chronic kidney disease

KDIGO provide a definition of CKD, as follows:

"CKD is defined as abnormalities of kidney structure or function, present for >3 months, with implications for health." This definition is based on various criteria, such as quantification of albuminuria, abnormal renal histology and structural abnormalities on kidney imaging. However, the staging of CKD is based on measuring the glomerular filtration rate (GFR).

**KDIGO staging of CKD**	
**Category of CKD**	**GFR (ml/min/1.73 m^2)**
G1 (normal or high)	≥90
G2 (mildly decreased – depending on age)	60-89
G3a	45-59
G3b	30-44
G4	15-29
G5	<15

Quantification of albuminuria is also used to predict prognosis of CDK:

**KDIGO staging of albuminuria**	
**Category of albuminuria**	**Albumin-creatinine ration (mg/g)**
A1	<30
A2	30-300
A3	>300

CKD has never been used as a primary or secondary outcome measure in any snakebite randomised clinical trial, which is probably due to the challenges of following up participants for 3 months. A consideration in favour of following up participants with abnormal renal function to 3 months, is that this is recommended clinical practise. This because people who develop CKD can benefit from secondary prevention, such as commencing an ACE inhibitor.

Requirement for renal replacement therapy

Requirement for renal replacement (RRT) is a common complication of snakebite in certain settings. An outcome measure based on the proportion of participants commencing RRT is of clear clinical significance. However, the reproducibility of this outcome measure should be considered. Firstly, most rural settings in LMICs do not have access to RRT and it may be necessary to define an outcome based on the point at which RRT is indicated, rather than commenced.

Secondly, (to quote the ADQI working group on clinical endpoints in RCTs in acute renal failure) "there is no consensus regarding the optimal timing for initiation of renal replacement therapy or specific criteria for the discontinuation of therapy. If the initiation or discontinuation of renal support is used as a clinical endpoint, strict criteria for these clinical decisions must be defined as part of the study design."

Based on a recent systematic review[21], clinical indications for renal replacement therapy in acute kidney injury include: refractory hyperkalaemia; fluid overload; acidosis; uraemia; and persistent oligo-anuria. The thresholds for defining each of these indications has varied between trials, for example hyperkalaemia has been defined as >6 mmol/l or >6.5 mmol/L.[21]

Major adverse kidney events[22]

A recent review has advocated for the use of clinically meaningful renal endpoints in clinical trials.[22] They coined the term 'MAKE', and suggested this composite outcome be used as a primary outcome in phase III clinical trials.

The major adverse kidney event (MAKE) composite outcome includes death, new dialysis requirement, or a reduction of eGFR of ≥25%. It can be measured at 30, 60 or 90 days, although the authors suggest measurement at 90 days is most valid, as it indicates irreversible CKD.[22]

Consensus of renal endpoints in peri-operative clinical trials

On searching the literature for core outcome sets in acute renal injury, one was identified that was developed by the StEP initiative for use in peri-operative clinical trials.[23] There are some similarities between renal injury secondary to peri-operative complications and snakebite envenoming. In particular, they involve an insult with an acute and known onset time, and they have a multifactorial aetiology that can include hypotension, blood loss, and direct nephrotoxicity (due to nephrotoxic medications or nephrotoxic venom components, respectively). Four key outcome measures were identified for use in future clinical trials:

1a) AKI: defined by the current KDIGO criteria (creatinine, oliguria, or initiation of RRT criteria)
1b) AKI: defined by the current KDIGO criteria, excluding oliguric criteria
2) Acute kidney disease $(AKD)_{Cr30}$: defined by ≥30% decline in estimated glomerular filtration rate (eGFR) from baseline at 30 days after operation in a patient who previously met creatinine-based KDIGO criteria for AKI within 7 days of surgery
3) Death or RRT: within a clearly defined time interval, preferably 30 or 90 days, after surgery
4) MAKE (mortality or RRT of any duration or ≥30% decline in eGFR from baseline measured at a pre-specified time, e.g. 30 or 90 days after operation).

Local tissue damage outcome measures

Summary results from systematic review of snakebite RCTs and protocols of ongoing RCTs		
**Outcome measure**	**Number of unique methods for recording the outcome measure**	**Number of studies using the outcome measure**
Development of skin blistering or necrosis	4	9
Swelling measured by circumference of bitten limb	3	9
Pain - ordinal scale	3	4
Skin and soft tissue infection	4	4
Opioid requirement	2	3
Swelling measured as proximal extension	1	3
Swelling measured by limb volume	1	2
Need for amputation, skin grafting or debridement	3	1

Measures of limb swelling

This was measured by circumference, length, or volume in 11 of the 58 snakebite clinical trials or clinical trial protocols. Nine of these 11 studies measured limb circumference. Although limb swelling is probably the most frequently occurring effect of envenoming, it is of limited clinical significance and the majority of patients are likely to recover without long-standing disability. In a placebo-controlled trial of antivenom for treating green pit viper bites with swelling, but without systemic envenoming, treatment was shown to significantly reduce limb circumference.[24] Yet the authors concluded that the finding was 'not clinically significant,' and advised against using antivenom for this purpose, in light of its cost and risk of adverse events.

Measures of skin blistering

Skin blistering occurs early in snakebite envenoming and is often a precursor to the development of necrotic lesions. A concern with using skin blistering as an outcome measure, is that any prospective treatment is likely to be administered at a time when separation of tissue layers due to the local effects of venom has already begun, and the likelihood of this outcome measure differentiating an effect are low.

Measures of skin necrosis

Skin necrosis can be a serious complication of local envenoming and can be particularly challenging to treat in rural LMIC settings. As for skin blistering, there is some concern that the pathological process of local tissue damage occurs too rapidly to be prevented by therapeutics, but necrosis does tend to occur later than blistering.

As well as capturing the numbers of participants with necrosis, the extent of necrosis is highly variable and is of clinical significance. There are various approaches to measuring the size of skin lesions. In burns, the percentage of body surface area is calculated, but this method is more suited to large lesions involving more than 10% of body surface area. Measurement of the length and width of lesions using measuring tape is feasible, although calculating the surface area of irregularly shaped lesions would not be possible. Photography of lesions alongside a paper ruler offers various advantages. It is quick for research staff to undertake. The image is stored and can be reviewed by clinicians with experience of assessing necrotic lesions. And software is available to accurately calculate the surface area of irregular lesions, as demonstrated below.[25]

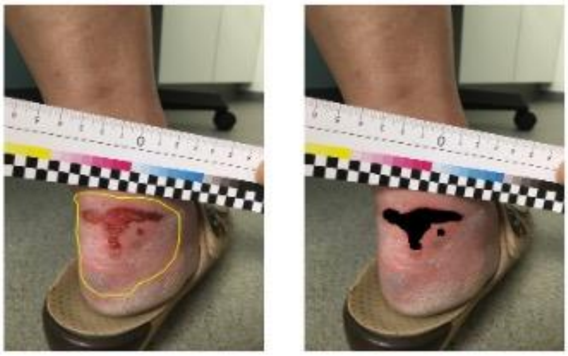

Numbers requiring amputation, skin grafting, fasciotomy and debridement

Requirement for surgery is of clear clinical significance. Yet there has only been one snakebite clinical trial which has used this outcome measure. This probably reflects the small sample sizes of snakebite clinical trials to date, as requirement for surgery is relatively uncommon.

Ensuring consistence in the use of surgical techniques would be important as the decision of whether to debride or to undertake fasciotomy is often contentious. In the context of a clinical trial, it might be feasible for participants with skin necrosis to have a virtual review by a trial plastic surgeon. This would standardise decisions to undertake surgery, as well as provide clinical oversight.

Patient-centred outcomes

Patient-centred outcomes will be of great importance for assessing for meaningful improvements in local tissue damage. Local tissue damage is undoubtedly the leading cause of disability amongst people with snakebite, and this can have serious consequences for an individual's ability to work or care for themself. Our systematic review found that no patient-centred outcome for snakebite trials have been used outside of the USA. The development of patient-centred outcomes requires patient-involvement and validation in an LMIC setting, which is outside the scope of the present study. This should form follow-on work from this study.

Systemic myotoxicity outcome measures

Summary results from systematic review of snakebite RCTs and protocols of ongoing RCTs		
**Outcome measure**	**Number of unique methods for recording the outcome measure**	**Number of studies using the outcome measure**
Creatine kinase	1	6
Myoglobinuria	2	2
Myalgia	1	1

Overview of myotoxicity in snakebite

In this section, we are considering myotoxicity (or rhabdomyolysis) as a systemic effect which is associated with a risk of renal injury, rather than local muscle damage at the bite site.

Sea snakes, certain rattlesnakes and some Russell's vipers can induce rhabdomyloysis, which can result in renal failure. In severe cases of sea snake envenoming, muscle weakness may ensue, and this can lead to respiratory failure.

Kidney function-based outcome measures (such as definitions of AKI, AKD and CKD), is described in the relevant section of this document. Renal injury is often multifactorial in aetiology, and it may be beneficial to include a specific measure of rhabdomyolysis, as detailed below.

Serum creatine kinase

Creatine kinase is an enzyme that originates in skeletal and cardiac muscle. If muscle breakdown occurs, it can be detected and quantified in the serum, and higher levels are associated with a risk of renal injury. Two published clinical trials[26,27] and four study protocols of incomplete snakebite trials have included measurement of CK. Both published trials examined people with *Bothrops* spp envenoming and the rise in CK was modest and not of clinical significance. In the cases with the most severe envenoming the CK ranged between 300-400 U/L,[27] which is a level unlikely to result in renal injury.

There has never been a randomised controlled trial of sea snake envenoming as these bites occur sporadically and in low numbers.

If CK were included as a core outcome measure, it would be important to consider the timing of measurement. CK will often peak in the first 48 hours, then gradually fall with supportive treatment. Measuring CK daily and calculating the area under the curve provides a measure of cumulative exposure to CK. Alternatively, the peak CK could be reported.

Myoglobinuria

Myoglobinuria can be measured using a bedside dip test. This measure is not quantifiable, and it would not be possible to identify participants with a clinically meaningful rise in myoglobin.

Myalgia

Assessing myalgia is non-specific for identifying clinically meaningful rhabdomyolysis.

Disability scales

Eight disability scales were identified from snakebite trials; all of which were conducted in the USA. Two of these scales were incompletely defined ('American Academy of Orthopaedic Surgeons (AAOS) normative outcome score' and the 'American Medical Association (AMA) disability rating score') and will not be considered for inclusion in the core outcome set. In addition to the six disability scales used in USA based snakebite trials, the World Health Organization Disability Assessment Schedule (WHODAS) will also be considered. The WHODAS has been validated in South America, Africa and Asia; is cross-cultural; is available in 30 languages; and assesses the physical, emotional and social impact of disease.

Disabilities of the arm, shoulder, and hand score (DASH score)

Task	No difficulty	Mild difficulty	Moderate difficulty	Severe difficulty	Unable
1. Open a tight new jar	1	2	3	4	5
2. Write	1	2	3	4	5
3. Turn a key	1	2	3	4	5
4. Prepare a meal	1	2	3	4	5
5. Push open a heavy door	1	2	3	4	5
6. Place an object on a shelf above your head	1	2	3	4	5
7. Do heavy household chores (e.g., wash floors)	1	2	3	4	5
8. Garden or do yard work	1	2	3	4	5
9. Make a bed	1	2	3	4	5
10. Carrying a shopping bag or briefcase	1	2	3	4	5
11. Carry a heavy object (> 10 lbs)	1	2	3	4	5
12. Change a lightbulb overhead	1	2	3	4	5
13. Wash or blow dry your hair	1	2	3	4	5
14. Wash your back	1	2	3	4	5
15. Put on a pullover sweater	1	2	3	4	5
16. Use a knife to cut food	1	2	3	4	5
17. Recreational activities which require little effort (e.g., cardplaying, knitting, etc.)	1	2	3	4	5
18. Recreational activities in which you take some force or impact through your arm, shoulder or hand (e.g., golf, hammering, tennis, etc.)	1	2	3	4	5
19. Recreational activities in which you move your arm freely (e.g., playing frisbee, badminton, etc.)	1	2	3	4	5
20. Manage transportation needs (getting from one place to another)	1	2	3	4	5
21. Sexual activities	1	2	3	4	5
22. During the past week, to what extent has your arm, shoulder or hand problem interfered with your normal social activities with family, friends, neighbours or groups? (circle number)	1	2	3	4	5
23. During the past week, were you limited in your work or other regular daily activities as a result of	1	2	3	4	5

your arm, shoulder or hand problem?					
24. Arm, shoulder or hand pain	1	2	3	4	5
25. Arm, shoulder or hand pain when you performed any specific activity	1	2	3	4	5
26. Tingling (pins and needles) in your arm, shoulder or hand	1	2	3	4	5
27. Weakness in your arm, shoulder or hand	1	2	3	4	5
28. Stiffness in your arm, shoulder or hand	1	2	3	4	5
29. During the past week, how much difficulty have you had sleeping because of the pain in your arm, shoulder or hand?	1	2	3	4	5
30. I feel less capable, less confident or less useful because of my arm, shoulder or hand problem	1	2	3	4	5

DASH disability/symptom score = ([(sum of n responses / n) - 1] x 25,where n is the number of completed responses)

Lower extremity functional scale (LEMS)

We are interested in knowing whether you are having any difficulty at all with the activities listed below because of your lower limb problem for which you are currently seeking attention. Please provide an answer for each activity.

Today, do you or would you have any difficulty at all with:

**Activities**	**Extreme Difficulty or Unable to Perform Activity**	**Quite a bit of difficulty**	**Moderate difficulty**	**A little bit of difficulty**	**No difficulty**
1. Any of your usual work, housework, or school activities	0	1	2	3	4
2. Your usual hobbies, recreational or sporting activities	0	1	2	3	4
3. Getting into or out of the bath	0	1	2	3	4
4. Walking between rooms	0	1	2	3	4
5. Putting on your shoes or socks	0	1	2	3	4
6. Squatting	0	1	2	3	4
7. Lifting an object, like a bag of groceries from the floor	0	1	2	3	4
8. Performing light activities around your home	0	1	2	3	4
9. Performing heavy activities around your home	0	1	2	3	4
10. Getting into or out of a car	0	1	2	3	4
11. Walking 2 blocks	0	1	2	3	4
12. Walking a mile	0	1	2	3	4
13. Going up or down 10 stairs (about 1 flight of stairs)	0	1	2	3	4
14. Standing for 1 hour	0	1	2	3	4
15. Sitting for 1 hour	0	1	2	3	4
16. Running on even ground	0	1	2	3	4
17. Running on uneven ground	0	1	2	3	4
18. Making sharp turns while running fast	0	1	2	3	4
19. Hopping	0	1	2	3	4
20. Rolling over in bed	0	1	2	3	4

Patient-reported outcome measurement information system physical function-10 score (PROMIS PF-10)

	No impairment	Mild impairment	Moderate impairment	Severe impairment	Unable to complete task
1. Are you able to dress yourself, including tying shoelaces and doing buttons?					
2. Are you able to shampoo your hair?					
3. Are you able to wash and dry your body?					
4. Are you able to get on and off the toilet?					
5. Does your health now limit you in doing vigorous activities such as running, lifting heavy objects, or participating in strenuous sports?					
6. Does your health now limit you in walking more than a mile?					
7. Does your health now limit you in climbing one flight of stairs?					
8. Does your health now limit you in lifting or carrying groceries?					
9. Does your health now limit you in bending, kneeling or stooping?					
10. Are you able to do chores such as vacuuming or yard work?					

Patient-specific functional scale (PSFS)

Read at baseline assessment: I am going to ask you to identify up to 3 important activities that you are unable to do or have difficulty with as a result of your problem.

1)
2)
3)

Read at baseline and at follow-up assessments: Today, are there any activities that you are unable to do or have difficulty with because of your problem? (show scale)

Scale:

0	1	2	3	4	5	6	7	8	9	10
Unable to perform									Able to perform activity at pre-injury levels	

Score diary:

Activity	Date/score								
1									
2									
3									

Patient's global impression of change-1 instrument

Since beginning treatment, how would you describe the change (if any) in activity limitations, symptoms, emotions and overall quality of life, related to your painful condition? (tick one box)

No change (or condition has got worse)	1
Almost the same, hardly any change at all	2
A little better, but no noticeable change	3
Somewhat better, but the change has not made any real difference	4
Moderately better, and a slight but noticeable change	5
Better, and a definite improvement that has made a real and worthwhile difference	6
A great deal better, and a considerable improvement that has made all the difference	7

The physical function domain of the SF-36 questionnaire

Task	Limited a lot	Limited a little	Not limited at all
Vigorous activities (running, lifting heavy objects, or sport)			
Moderate activities (moving a table, pushing vacuum cleaner, or playing golf)			
Lifting or carrying groceries			
Climbing several flights of stairs			
Climbing one flight of stairs			
Bending, kneeling, or stooping			
Walking more than a mile			
Walking several blocks			
Walking one block			
Bathing or dressing yourself			

World Health Organization Disability Assessment Schedule (WHODAS)

https://www.who.int/classifications/icf/WHODAS2.0_12itemsSELF.pdf

(12-item self-administered version)

This questionnaire asks about difficulties due to health conditions. Health conditions include diseases or illnesses, other health problems that may be short or long lasting, injuries, mental or emotional problems, and problems with alcohol or drugs.

Think back over the past 30 days and answer these questions, thinking about how much difficulty you had doing the following activities. For each question, please circle only one response.

In the past 30 days, how much difficulty did you have in:						
S1	Standing for long periods such as 30 minutes?	None	Mild	Moderate	Severe	Extreme or cannot do
S2	Taking care of your household responsibilities?	None	Mild	Moderate	Severe	Extreme or cannot do
S3	Learning a new task, for example, learning how to get to a new place?	None	Mild	Moderate	Severe	Extreme or cannot do
S4	How much of a problem did you have joining in community activities (for example, festivities, religious or other activities) in the same way as anyone else can?	None	Mild	Moderate	Severe	Extreme or cannot do
S5	How much have you been emotionally affected by your health problems?	None	Mild	Moderate	Severe	Extreme or cannot do
S6	Concentrating on doing something for ten minutes?	None	Mild	Moderate	Severe	Extreme or cannot do
S7	Walking a long distance such as a kilometre [or equivalent]?	None	Mild	Moderate	Severe	Extreme or cannot do
S8	Washing your whole body?	None	Mild	Moderate	Severe	Extreme or cannot do
S9	Getting dressed?	None	Mild	Moderate	Severe	Extreme or cannot do
S10	Dealing with people you do not know?	None	Mild	Moderate	Severe	Extreme or cannot do
S11	Maintaining a friendship?	None	Mild	Moderate	Severe	Extreme or cannot do
S12	Your day-to-day work?	None	Mild	Moderate	Severe	Extreme or cannot do

H1	Overall, in the past 30 days, how many days were these difficulties present?	Record number of days ____
H2	In the past 30 days, for how many days were you totally unable to carry out your usual activities or work because of any health condition?	Record number of days ____
H3	In the past 30 days, not counting the days that you were totally unable, for how many days did you cut back or reduce your usual activities or work because of any health condition?	Record number of days ____

Adverse event outcome measures

Adverse event reporting amongst snakebite trials from our systematic review

No adverse events were reported in 39.3% of snakebite trials. The majority of trials did not provide a definition for anaphylaxis or late serum sickness. Follow-up of participants was invariably too short to identify late serum sickness. Anaphylaxis was defined based on published criteria in 8·6% of studies. No studies used published criteria to define late serum sickness.

Anaphylaxis

As antivenoms contains animal derived antibody or antibody fragments, they can induce an acute hypersensitivity reaction. In a clinical trial based in Sri Lanka, 75% of participants had an acute reaction to antivenom, and 43% of these reactions were classified as severe.[28]

Rates of anaphylaxis need to be consistently reported, so that the safety of antivenom products can be compared. Defined published criteria for defining anaphylaxis were only followed in two published clinical trials and three protocols of incomplete clinical trials in snakebite. Four of these studies defined anaphylaxis according to the National Institute of Allergy and Infectious Disease/Food Allergy and Anaphylaxis Network (NIAID-FAAN) consensus criteria and assessed severity using the Brown grading system.[29–32] One study protocol of an ongoing trial defined anaphylaxis according to the European Academy of Allergy and Immunology criteria – which are identical to the NIAID-FAAN definition.[33]

**NIAID-FAAN consensus criteria for defining anaphylaxis:**[34]	
Anaphylaxis is highly likely when any one of the following 3 criteria are fulfilled:	
1	Acute onset of an illness (minutes to several hours) with involvement of the skin, mucosal tissue, or both (e.g., generalized hives, pruritus or flushing, swollen lips-tongue-uvula)
AND at least one of:	
a)	Respiratory compromise (eg, dyspnea, wheeze-bronchospasm, stridor, reduced PEF, hypoxemia)
b)	Reduced BP or associated symptoms of end-organ dysfunction (eg, hypotonia [collapse], syncope, incontinence)
2	Two or more of the following that occur rapidly after exposure to a likely allergen for that patient (minutes to several hours):
a)	Involvement of the skin-mucosal tissue (eg, generalized hives, itch-flush, swollen lips-tongue-uvula)
b)	Respiratory compromise (eg, dyspnea, wheeze-bronchospasm, stridor, reduced PEF, hypoxemia)
c)	Reduced BP or associated symptoms (eg, hypotonia [collapse], syncope, incontinence)
d)	Persistent gastrointestinal symptoms (eg, crampy abdominal pain, vomiting)
3	Reduced BP after exposure to known allergen for that patient (minutes to several hours):
a)	Infants and children: low systolic BP (age specific) or greater than 30% decrease in systolic BP
b)	Adults: systolic BP of less than 90 mm Hg or greater than 30% decrease from that person's baseline

Brown criteria for severity grading anaphylaxis[35]	
1) Mild (skin and subcutaneous tissues only)*	Generalized erythema, urticaria, periorbital oedema, or angioedema
2) Moderate (features suggesting respiratory, cardiovascular, or gastrointestinal involvement)	Dyspnoea, stridor, wheeze, nausea, vomiting, dizziness (presyncope), diaphoresis, chest or throat tightness, or abdominal pain
3) Severe (hypoxia, hypotension, or neurologic compromise)	Cyanosis or SpO2 ≤92% at any stage, hypotension (SBP < 90mmHgin adults), confusion, collapse, LOC, or incontinence

SBP, Systolic blood pressure; LOC, loss of consciousness.
*Mild reactions can be further subclassified into those with and without angioedema

Serum sickness

Serum sickness is a delayed (type III) hypersensitivity reaction due to an IgG mediated immune response to the animal proteins in antivenom. It typically presents with fever, rash and arthralgia. It has a delayed onset and

the short follow-up of many clinical trials of snakebite means that it is under-reported. In the Australian Snakebite Project (ASP) 29% of antivenom recipients developed serum sickness.[36]

There is a lack of consensus criteria for the diagnosis of serum sickness. The ASP study defined serum sickness as follows.[36]

**ASP serum sickness definition**[36]
≥3 of the following clinical features starting 5–20 days after antivenom administration:
• Fever
• Erythematous rash or urticaria
• Myalgia or arthralgia
• Headache
• Malaise
• Nausea or vomiting

Information Pack References

1. Blackwood B, Ringrow S, Clarke M, et al. A Core Outcome Set for Critical Care Ventilation Trials. *Crit Care Med*. 2019;47(10):1324-1331. doi:10.1097/CCM.0000000000003904

2. Lawn ND, Fletcher DD, Henderson RD, Wolter TD, Wijdicks EFM. Anticipating Mechanical Ventilation in Guillain-Barré Syndrome. *Arch Neurol*. 2001;58(6):893. doi:10.1001/archneur.58.6.893

3. Pontoppidan H, Geffin B, Lowenstein E. Acute Respiratory Failure in the Adult. http://dx.doi.org/10.1056/NEJM197210192871605. doi:10.1056/NEJM197210192871605

4. Blackwood B, Clarke M, McAuley DF, McGuigan PJ, Marshall JC, Rose L. How outcomes are defined in clinical trials of mechanically ventilated adults and children. *Am J Respir Crit Care Med*. 2014;189(8):886-893. doi:10.1164/rccm.201309-1645PP

5. Yunusova Y, Plowman EK, Green JR, Barnett C, Bede P. Clinical Measures of Bulbar Dysfunction in ALS. *Front Neurol*. 2019;10. doi:10.3389/fneur.2019.00106

6. Hughes RA, Swan AV, van Doorn PA. Intravenous immunoglobulin for Guillain-Barré syndrome. *Cochrane Database Syst Rev*. 2014;2014(9). doi:10.1002/14651858.CD002063.pub6

7. Randomised trial of plasma exchange, intravenous immunoglobulin, and combined treatments in Guillain-Barré syndrome. Plasma Exchange/Sandoglobulin Guillain-Barré Syndrome Trial Group. *Lancet*. 1997;349(9047):225-230.

8. Isbister GK. Snakebite doesn't cause disseminated intravascular coagulation: coagulopathy and thrombotic microangiopathy in snake envenoming. *Semin Thromb Hemost*. 2010;36(4):444-451. doi:10.1055/s-0030-1254053

9. Thomas PP, Jacob J. Randomised trial of antivenom in snake envenomation with prolonged clotting time. *Br Med J (Clin Res Ed)*. 1985;291(6489):177-178. doi:10.1136/bmj.291.6489.177

10. Paul V, Pudoor A, Earali J, John B, Anil Kumar CS, Anthony T. Trial of low molecular weight heparin in the treatment of viper bites. *J Assoc Physicians India*. 2007;55:338-342.

11. de Brito Sousa JD, Sachett JAG, de Oliveira SS, et al. Accuracy of the Lee–White Clotting Time Performed in the Hospital Routine to Detect Coagulopathy in Bothrops atrox Envenomation. *Am J Trop Med Hyg*. 2018;98(5):1547-1551. doi:10.4269/ajtmh.17-0992

12. Isbister GK, Williams V, Brown SGA, White J, Currie BJ. Clinically applicable laboratory end-points for treating snakebite coagulopathy. *Pathology*. 2006;38(6):568-572. doi:10.1080/00313020601024045

13. Schulman S, Kearon C, Subcommittee on Control of Anticoagulation of the Scientific and Standardization Committee of the International Society on Thrombosis and Haemostasis. Definition of major bleeding in clinical investigations of antihemostatic medicinal products in non-surgical patients. *J Thromb Haemost*. 2005;3(4):692-694. doi:10.1111/j.1538-7836.2005.01204.x

14. Nijboer JMM, van der Horst ICC, Hendriks HGD, ten Duis H-J, Nijsten MWN. Myth or reality: hematocrit and hemoglobin differ in trauma. *J Trauma*. 2007;62(5):1310-1312. doi:10.1097/TA.0b013e3180341f54

15. Guideline on clinical investigation of medicinal products for the treatment of venous thromboembolic disease. :21.

16. Jetton JG, Boohaker LJ, Sethi SK, et al. Incidence and outcomes of neonatal acute kidney injury (AWAKEN): a multicentre, multinational, observational cohort study. *The Lancet Child & Adolescent Health*. 2017;1(3):184-194. doi:10.1016/S2352-4642(17)30069-X

17. Kidney Disease: Improving Global Outcomes (KDIGO) Acute Kidney Injury Work Group. KDIGO Clinical Practice Guideline for Acute Kidney Injury. *Kidney inter, Suppl*. 2012;2(1):1-138. doi:10.1038/kisup.2012.1

18. Levi TM, de Souza SP, de Magalhães JG, et al. Comparison of the RIFLE, AKIN and KDIGO criteria to predict mortality in critically ill patients. *Rev Bras Ter Intensiva*. 2013;25(4):290-296. doi:10.5935/0103-507X.20130050

19. Grams ME, Sang Y, Coresh J, et al. Candidate Surrogate End Points for ESRD after AKI. *J Am Soc Nephrol*. 2016;27(9):2851-2859. doi:10.1681/ASN.2015070829

20. Chawla LS, Bellomo R, Bihorac A, et al. Acute kidney disease and renal recovery: consensus report of the Acute Disease Quality Initiative (ADQI) 16 Workgroup. *Nat Rev Nephrol*. 2017;13(4):241-257. doi:10.1038/nrneph.2017.2

21. Gaudry S, Hajage D, Benichou N, et al. Delayed versus early initiation of renal replacement therapy for severe acute kidney injury: a systematic review and individual patient data meta-analysis of randomised clinical trials. *The Lancet*. 2020;395(10235):1506-1515. doi:10.1016/S0140-6736(20)30531-6

22. Billings FT, Shaw AD. Clinical trial endpoints in acute kidney injury. *Nephron Clin Pract*. 2014;127(1-4):89-93. doi:10.1159/000363725

23. McIlroy DR, Bellomo R, Billings FT, et al. Systematic review and consensus definitions for the Standardised Endpoints in Perioperative Medicine (StEP) initiative: renal endpoints. *British Journal of Anaesthesia*. 2018;121(5):1013-1024. doi:10.1016/j.bja.2018.08.010

24. Rojnuckarin P, Chanthawibun W, Noiphrom J, Pakmanee N, Intragumtornchai T. A randomized, double-blind, placebo-controlled trial of antivenom for local effects of green pit viper bites. *Transactions of the Royal Society of Tropical Medicine and Hygiene*. 2006;100(9):879-884. doi:10.1016/j.trstmh.2005.10.006

25. Mirzaalian-Dastjerdi H, Töpfer D, Bangemann M, Maier A. Detecting and Measuring Surface Area of Skin Lesions. In: Maier A, Deserno TM, Handels H, Maier-Hein KH, Palm C, Tolxdorff T, eds. *Bildverarbeitung für die Medizin 2018*. Informatik aktuell. Springer; 2018:29-34. doi:10.1007/978-3-662-56537-7_20

26. Mendonça-da-Silva I, Magela Tavares A, Sachett J, et al. Safety and efficacy of a freeze-dried trivalent antivenom for snakebites in the Brazilian Amazon: An open randomized controlled phase IIb clinical trial. Calvete JJ, ed. *PLOS Neglected Tropical Diseases*. 2017;11(11):e0006068. doi:10.1371/journal.pntd.0006068

27. Otero-Patiño R, Segura A, Herrera M, et al. Comparative study of the efficacy and safety of two polyvalent, caprylic acid fractionated [IgG and F(ab')2] antivenoms, in Bothrops asper bites in Colombia. *Toxicon*. 2012;59(2):344-355. doi:10.1016/j.toxicon.2011.11.017

28. Silva HA de, Pathmeswaran A, Ranasinha CD, et al. Low-Dose Adrenaline, Promethazine, and Hydrocortisone in the Prevention of Acute Adverse Reactions to Antivenom following Snakebite: A Randomised, Double-Blind, Placebo-Controlled Trial. *PLOS Medicine*. 2011;8(5):e1000435. doi:10.1371/journal.pmed.1000435

29. Isbister GK. *Randomised Controlled Trial of Fresh Frozen Plasma to Speed the Recovering of Venom Induced Consumption Coagulopathy in Patients with Russell's Viper Envenoming in Sri Lanka*.; 2008. Accessed January 19, 2021. https://www.anzctr.org.au/Trial/Registration/TrialReview.aspx?id=83196

30. Isbister GK. *A Multicentre Double-Blind Randomised Placebo-Controlled Trial of Early Antivenom versus Placebo in the Treatment of Red Bellied Black Snake Envenoming*.; 2011. Accessed January 19, 2021. https://www.anzctr.org.au/Trial/Registration/TrialReview.aspx?id=343001

31. Isbister GK, Buckley NA, Page CB, et al. A randomized controlled trial of fresh frozen plasma for treating venom-induced consumption coagulopathy in cases of Australian snakebite (ASP-18). *Journal of Thrombosis and Haemostasis*. 2013;11(7):1310-1318. doi:10.1111/jth.12218

32. Isbister GK, Jayamanne S, Mohamed F, et al. A randomized controlled trial of fresh frozen plasma for coagulopathy in Russell's viper (Daboia russelii) envenoming. *J Thromb Haemost*. 2017;15(4):645-654. doi:10.1111/jth.13628

33. Lamb T. *An Adaptive Clinical Trial to Determine the Optimal Initial Dose of Lyophilized, Species Specific Monovalent Antivenom for the Management of Systemic Envenoming by Daboia Siamensis (Eastern Russell's Viper) in Myanmar*. clinicaltrials.gov; 2020. Accessed January 18, 2021. https://clinicaltrials.gov/ct2/show/NCT04210141

34. Sampson HA, Muñoz-Furlong A, Campbell RL, et al. Second symposium on the definition and management of anaphylaxis: Summary report—Second National Institute of Allergy and Infectious Disease/Food Allergy and Anaphylaxis Network symposium. *Journal of Allergy and Clinical Immunology*. 2006;117(2):391-397. doi:10.1016/j.jaci.2005.12.1303

35. Brown SGA. Clinical features and severity grading of anaphylaxis. *J Allergy Clin Immunol*. 2004;114(2):371-376. doi:10.1016/j.jaci.2004.04.029

36. Ryan NM, Kearney RT, Brown SGA, Isbister GK. Incidence of serum sickness after the administration of Australian snake antivenom (ASP-22). *Clinical Toxicology*. 2016;54(1):27-33. doi:10.3109/15563650.2015.1101771

Overview of the Shortlisting Questionnaire Scores and the Consensus Meeting Vote Results on Potential Core Outcome Measures

		**Screening questionnaire results**				**Consensus meeting results**		
**Outcome domain**	**Group**	**Mean score**	**Essential (%)**	**Desirable (%)**	**Inappropriate (%)**	**Selected for consideration by consensus group**	**Consensus group percentage voted in favour**	**Selected for inclusion in the COS**
All-cause mortality	Mortality	7.8	80	15	5			
Cause specific mortality	Mortality	6.3	55	30	15			
Mortality with community follow-up	Mortality	7.1	60	35	5	Yes	100%	Yes
In-hospital mortality only	Mortality	5.8	45	25	30			
WHODAS	Disability scale	6.6	50	35	0	Yes	100%	Yes
PSFS	Disability scale	6.4	45	40	10	Yes	90%	Yes
PROMIS	Disability scale	5.3	20	55	15			
PGICI	Disability scale	5.2	25	45	20			
DASH	Disability scale	4.9	15	50	25			
LEFS	Disability scale	4.9	10	50	30			
SF36	Disability scale	4.5	10	50	35			
Requirement for invasive ventilation	Neurotoxicity	8.1	90	10	0	Yes	100%	Yes
Duration of invasive ventilation	Neurotoxicity	7.2	65	30	5	Yes	100%	Yes
Ptosis	Neurotoxicity	6.0	45	35	20	Yes	33%	
Assessment of functional ability (similar to Guillain-Barré syndrome scale)	Neurotoxicity	6.0	35	60	5			

Extraocular muscle palsy	Neurotoxicity	5.2	30	45	25			
Bulbar palsy	Neurotoxicity	5.2	20	55	20			
Spirometry	Neurotoxicity	4.8	20	50	30			
Measures of non-facial muscle weakness	Neurotoxicity	4.3	10	60	30			
Electromyography	Neurotoxicity	3.7	0	55	45			
ISTH defined major bleeding	Haemorrhage	7.4	75	20	5	Yes	100%	Yes
EMA definition of clinically relevant non-major bleeding	Haemorrhage	6.4	55	30	10	Yes	91%	Yes
Blood transfusion requirement	Haemorrhage	6.2	50	40	10			
Haemoglobin	Haemorrhage	6.0	40	40	20			
Bleeding index	Haemorrhage	5.9	30	45	20			
Time to cessation of non-major bleeding events	Haemorrhage	5.6	35	30	30			
Reticulocyte count	Haemorrhage	3.4	0	35	50			
20-minute whole blood clotting test	Coagulopathy	7.1	65	20	10	Yes	22%	
INR lab	Coagulopathy	6.7	50	45	5	Yes	90%	Yes
Prothrombin time	Coagulopathy	6.3	50	40	10			
Activated partial thromboplastin time	Coagulopathy	5.7	40	45	15			
Point of care INR assay	Coagulopathy	5.5	25	60	10			
Platelet count	Coagulopathy	5.5	30	45	20			
Fibrinogen	Coagulopathy	5.2	25	50	25			
Lee White clotting time	Coagulopathy	4.6	10	50	30			
Fibrinogen degradation products including D-dimer	Coagulopathy	4.3	5	55	35			
Thromboelastography	Coagulopathy	3.9	0	50	45			

Clotting factor transfusion requirement	Coagulopathy	3.8	10	30	55			
Clotting factors	Coagulopathy	3.5	0	50	50			
Local effects requiring surgery	Local tissue damage	7.2	75	5	15	Yes	87%	Yes
Disability scale to measure local tissue damage	Local tissue damage	6.8	50	50	0			
Ordinal pain scale	Local tissue damage	6.2	40	40	15	Yes	27%	
Surface area skin necrosis using digital technology	Local tissue damage	6.0	30	60	5	Yes	71%	Yes
-Require digital technology?	Local tissue damage	NA	NA	NA	NA	Yes	60%	
Creatine kinase to measure local myonecrosis	Local tissue damage	5.5	30	40	25			
Limb proximal extension	Local tissue damage	5.3	25	40	25			
Number with skin or soft tissue infection	Local tissue damage	5.1	25	35	30			
Surface area skin necrosis using measuring tape	Local tissue damage	5.0	20	60	15			
Limb circumference	Local tissue damage	4.7	20	35	35			
Surface area of skin blisters	Local tissue damage	4.7	15	55	25			
Number with skin blisters	Local tissue damage	4.4	15	40	40			
Volume of limb swelling	Local tissue damage	4.4	10	45	35			
More than half limb swollen	Local tissue damage	4.3	10	50	30			
Opioid requirement	Local tissue damage	3.8	10	30	50			
KDIGO defined Acute kidney injury	Renal injury	6.9	55	20	5	Yes	64%	
Requirement for renal replacement therapy	Renal injury	6.2	40	20	20	Yes	92%	Yes
Acute Kidney Disease by non-KDIGO definitions	Renal injury	5.5	15	65	0			
Renal injury at 14 days	Renal injury	NA	NA	NA	NA	Yes	38%	

Renal injury at 42 days	Renal injury	NA	NA	NA	NA	Yes	77%	Yes
KDIGO defined chronic kidney disease	Renal injury	5.1	10	65	5	Yes	43%	
MAKE renal endpoints	Renal injury	4.9	10	50	15			
Renal peri-operative core outcome set	Renal injury	4.9	10	50	15			
Serum urea	Renal injury	4.6	25	10	45			
Albumin-to-Creatinine ratio	Renal injury	4.1	10	30	40			
Proteinuria dip	Renal injury	4.1	10	35	45			
Haemoglobinuria dip	Renal injury	4.0	10	35	40			
Area under the curve creatine kinase	Systemic myotoxicity	6.0	40	35	15	Yes	64%	
Renal endpoints to measure rhabdomyolysis	Systemic myotoxicity	6.0	40	35	15			
Peak creatine kinase	Systemic myotoxicity	5.4	30	35	25			
Myoglobinuria dip	Systemic myotoxicity	4.6	15	45	30			
Myalgia	Systemic myotoxicity	3.6	0	40	50			
NIAID definition anaphylaxis	Antivenom allergic reaction	7.0	60	25	5	Yes	0%	
ASP definition serum sickness	Antivenom allergic reaction	6.8	45	40	5	Yes	100%	Yes
Brown criteria grade anaphylaxis	Antivenom allergic reaction	6.3	50	35	5	Yes	73%	Yes
Include both NIAID and BROWN definitions	Antivenom allergic reaction	NA	NA	NA	NA	Yes	27%	
Hypotension	Cardiotoxicity	6.8	65	10	20	Yes	87%	Yes
hypotension - change	Cardiotoxicity	NA	NA	NA	NA			
hypotension - shock	Cardiotoxicity	NA	NA	NA	NA	Yes	100%	Yes

Additional antivenom dose	Venom/antivenom quantification	6.8	60	25	10		
Total antivenom dose	Venom/antivenom quantification	6.5	50	35	10		
Venom antigenaemia	Venom/antivenom quantification	4.2	5	50	30		
Duration of hospital stay	Others	7.0	60	35	0		
Return to work	Others	5.4	25	50	15		
Limb weakness	Others	5.1	15	55	25		
Gastrointestinal symptoms	Others	4.7	15	30	35		
Leucocyte count	Others	4.3	10	35	45		
Hypoxic brain injury	Others	4.2	15	25	45		
Lactate dehydrogenase	Others	3.8	5	30	55		
Number of physiotherapy appointments	Others	3.6	5	30	60		
Anosmia	Others	3.4	0	25	45		
Neutrophil Gelatinase Associated Lipocalin (NGAL)	Others	3.3	0	15	50		
Blood metalloproteinase quantification	Others	3.2	0	30	60		

DASH, Disabilities of the Arm, Shoulder and Hand outcome measure; EMA, European Medicines Agency; INR, International Normalised Ratio; ISTH, International Society on Thrombosis and Haemostasis; KDIGO, Kidney Disease Improving Global Outcomes; LEFS, Lower Extremity Functional Scale; MAKE, Major Adverse Kidney Events; NIAID, National Institute of Allergy and Infectious Diseases; PSFS, Patient Specific Functional Scale; PROMIS, Patient-Reported Outcome Measurement Information System; PGICI, Patient's Global Impression of Change scale; SF36, 36-item short-form; WHODAS, World Health Organization Disability Assessment Scale.

Outcome Measurement Instrument References

- Üstün, T. B. Measuring health and disability: Manual for WHO Disability Assessment Schedule WHODAS 2.0. 2010. https://www.who.int/publications/i/item/measuring-health-and-disability-manual-for-who-disability-assessment-schedule-(-whodas-2.0) (accessed Sept 25, 2021).

- Gerardo CJ, Vissoci JRN, de Oliveira LP, et al. The validity, reliability and minimal clinically important difference of the patient specific functional scale in snake envenomation. PLoS One 2019; 14. DOI:10.1371/journal.pone.0213077.

- Theophanous RG, Vissoci JRN, Wen FH, et al. Validity and reliability of telephone administration of the patient-specific functional scale for the assessment of recovery from snakebite envenomation. PLOS Neglected Tropical Diseases 2019; 13: e0007935.

- Patient-Reported Outcomes Measurement Information System. PROMIS Physical Function Short Form 10a. 2017. https://www.healthmeasures.net/administrator/components/com_instruments/uploads/PROMIS%20SF%20v2.0%20-%20Physical%20Function%2010a%203-28-2017.pdf (accessed Jan 14, 2022).

- Hurst H, Bolton J. Assessing the clinical significance of change scores recorded on subjective outcome measures. Journal of Manipulative and Physiological Therapeutics 2004; 27: 26–35.

- Beaton DE, Katz JN, Fossel AH, Wright JG, Tarasuk V, Bombardier C. Measuring the whole or the parts? Validity, reliability, and responsiveness of the Disabilities of the Arm, Shoulder and Hand outcome measure in different regions of the upper extremity. J Hand Ther 2001; 14: 128–46.

- Binkley JM, Stratford PW, Lott SA, Riddle DL. The Lower Extremity Functional Scale (LEFS): scale development, measurement properties, and clinical application. North American Orthopaedic Rehabilitation Research Network. Phys Ther 1999; 79: 371–83.

- Ware JE, Sherbourne CD. The MOS 36-item short-form health survey (SF-36). I. Conceptual framework and item selection. Med Care 1992; 30: 473–83.

- Hughes RA, Swan AV, van Doorn PA. Intravenous immunoglobulin for Guillain-Barré syndrome. Cochrane Database Syst Rev 2014; 2014. DOI:10.1002/14651858.CD002063.pub6.

- Schulman S, Kearon C, Subcommittee on Control of Anticoagulation of the Scientific and Standardization Committee of the International Society on Thrombosis and Haemostasis. Definition of major bleeding in clinical investigations of antihemostatic medicinal products in non-surgical patients. J Thromb Haemost 2005; 3: 692–4.

- European Medicines Agency. Guideline on clinical investigation of medicinal products for the treatment of venous thromboembolic disease. 2016. https://www.ema.europa.eu/en/documents/regulatory-procedural-guideline/guideline-clinical-investigation-medicinal-products-treatment-venous-thromboembolic-disease_en.pdf (accessed Sept 25, 2021).

- Poisoning by Bites of the Saw-Scaled or Carpet Viper (Echis carinatus) in Nigeria. QJM: An International Journal of Medicine 1977; published online Jan. DOI:10.1093/oxfordjournals.qjmed.a067493.

- Lee RI, White PD. A Clinical Study Of The Coagulation Time Of Blood. The American Journal of the Medical Sciences 1913; 145: 495–503.

- Kidney Disease: Improving Global Outcomes (KDIGO) Acute Kidney Injury Work Group. KDIGO Clinical Practice Guideline for Acute Kidney Injury. Kidney inter, Suppl 2012; 2: 1–138.

- Kidney Disease: Improving Global Outcomes (KDIGO). KDIGO Clinical Practice Guideline for the Evaluation and Management of Chronic Kidney Disease. 2013. https://kdigo.org/wp-content/uploads/2017/02/KDIGO_2012_CKD_GL.pdf (accessed Oct 2, 2020).

- Billings FT, Shaw AD. Clinical trial endpoints in acute kidney injury. Nephron Clin Pract 2014; 127: 89–93.

- McIlroy DR, Bellomo R, Billings FT, et al. Systematic review and consensus definitions for the Standardised Endpoints in Perioperative Medicine (StEP) initiative: renal endpoints. British Journal of Anaesthesia 2018; 121: 1013–24.

- Sampson HA, Muñoz-Furlong A, Campbell RL, et al. Second symposium on the definition and management of anaphylaxis: Summary report—Second National Institute of Allergy and Infectious Disease/Food Allergy and Anaphylaxis Network symposium. Journal of Allergy and Clinical Immunology 2006; 117: 391–7.

- Ryan NM, Kearney RT, Brown SGA, Isbister GK. Incidence of serum sickness after the administration of Australian snake antivenom (ASP-22). Clinical Toxicology 2016; 54: 27–33.

- Brown SGA. Clinical features and severity grading of anaphylaxis. J Allergy Clin Immunol 2004; 114: 371–6.

The Full Core Outcome Measurement Set

Universal outcome categories: for all snakebite clinical trials
**Mortality:**
All-cause mortality ☏<br><br>All-cause mortality measured at 42 days after randomisation (via telephone or face-to-face follow-up).
**Disability scales:**
WHODAS 2.0 ☏<br><br>WHO 12-item Disability Assessment Scale 2.0 measured at 42 days after randomisation (for children aged ≥2 and ≤17 years, use the WHODAS-child tool; do not measure WHODAS for children <2 years).
Patient-specific functional scale ☏<br><br>Patient Specific Functional Scale measured at day 14 and at day 42 after randomisation.
**Adverse events:**
Brown grading of allergic reactions<br><br>Proportion with mild, moderate, and severe allergic reactions according to the Brown grading system. Record the most severe grade of event occurring within the 6-hour period after randomisation.
ASP defined serum sickness ☏<br><br>Proportion with serum sickness defined according to the Australian Snakebite Project (ASP) definition. To streamline timing of assessments, this can be assessed at telephone or face-to-face follow-up at day 14 and day 42 after randomisation.

ASP definition of sickness:
≥3 of the following clinical features starting 5–20 days after randomisation:
• Fever
• Erythematous rash or urticaria
• Myalgia or arthralgia
• Arthritis
• Headache
• Malaise
• Nausea or vomiting

Syndrome specific outcome categories: for use depending on the biting species

Neurotoxic envenoming:

Need for intubation and ventilation

a) Proportion needing* intubation[ø] within 48 hours after randomisation

** In settings where resources for intubation are not readily available, the proportion of participants needing intubation should be reported, regardless of whether the intervention was implemented. As far as possible, the criteria for identifying a participant as needing intubation should be reported.*

ø Intubation includes insertion of an endotracheal tube or laryngeal mask airway.

b) Proportion needing mechanical or manual ventilation[†] within 48 hours after randomisation

*†Onset of ventilation defined as continuous use of bag valve mask ventilation or mechanical ventilation via an endotracheal tube or laryngeal mask airway. **

**Where ventilation equipment is not readily available, the proportion of participants needing ventilation should be reported, regardless of whether the intervention was implemented. As far as possible, the criteria for identifying a participant as needing ventilation should be reported.*

c) Duration of ventilation: Time from randomization[‡] until first successful unassisted breathing[¥] treating death before successful unassisted breathing as a competing risk, recorded until 42 days after randomisation.

Mean and median time (hours) of duration of ventilation is calculated based on the cumulative incidence of the outcome, treating death as a competing risk in all patients, and separately based on the probability of successful unassisted breathing conditional on remaining alive on ventilation. Median calculated directly from probabilities, mean calculated from area under the curve.

‡ For participants that are ventilated prior to randomisation, timing should start from the point of randomisation. For participants ventilated after randomisation, timing should start from the point of ventilation.

¥ Unassisted breathing is defined as no inspiratory support except for high-flow oxygen therapy or continuous positive airway pressure. Success is defined as remaining to breathe unassisted at 48 hours after ceasing respiratory support. The 48-hour success period is not included in the calculation of duration of ventilation.

Haemotoxic or coagulopathic envenoming:
ISTH defined major bleeding<br><br>Proportion with in-hospital major bleeding (defined according to the International Society on Thrombosis and Haemostasis criteria) in the 7-days following randomisation.

**ISTH definition of major bleeding events:**
• Fatal bleeding OR
• Symptomatic bleeding in a critical area or organ (such as intracranial, intraspinal, intraocular, retroperitoneal, intraarticular or pericardial, or intramuscular with compartment syndrome) OR
• Bleeding causing a fall in haemoglobin level of 2 g/dL (1.24 mmol/L) or more; or leading to transfusion of two or more units of whole blood or red cells.

Clinically relevant non-major bleeding (CRNMB)

a. **Early cessation of non-major bleeding:** amongst participants with active bleeding* at randomisation, proportion of participants with cessation of bleeding at six hours after randomisation.

** Active bleeding defined as epistaxis; gingival bleeding; or prolonged [>5 minutes] bleeding from a venepuncture site.*

Haematemesis, rectal bleeding, and frank haematuria are not included in the definition of early cessation of bleeding as it is not possible to distinguish whether the presence of blood from these sites represents active bleeding, or delayed transit of past bleeding.

b. **Delayed onset clinically relevant non-major bleeding:** proportion of participants developing clinically relevant non-major bleeding† between 6-48 hours after randomisation

† Clinically relevant non-major bleeding defined as epistaxis, gum bleeding, prolonged (>5 minutes) bleeding from a venepuncture site, haematemesis, rectal bleeding, or frank haematuria.

INR

Mean and median INR measured at 6 and 12-hours after randomisation using a laboratory assay or validated bedside INR meter

Local tissue damage:

Requirement for surgery ☏

Proportion requiring* debridement of non-viable tissue, skin grafting or amputation by day 42 after randomisation.

** In settings where surgery is not readily available, the proportion that are judged to require surgery (regardless of whether they received it) should be reported. The criteria for offering surgery should be standardised and clearly reported.*

Total surface area of skin necrosis

Mean and median total surface area† of full thickness skin necrosis* in cm^2 at 48 hours after randomisation. For each participant, all lesions associated with the snakebite should be measured and summed.

† *To better standardise measurement, particularly of irregularly shaped lesions, use of validated digital technology to analyse photographs is preferable, although not obligatory.*

* *Full thickness tissue loss is present when subcutaneous fat, muscle, tendon or bone are exposed.*

Renal injury or systemic myotoxicity:

Requirement for renal replacement therapy

Proportion of participants with a new onset requirement* for renal replacement therapy (RRT) – includes haemodialysis, haemofiltration or peritoneal dialysis – within 42 days of randomization

* *The criteria for initiating RRT should be reported in the protocol. In settings where RRT is not readily available, the proportion that would require RRT by specified criteria (regardless of whether they received it) should be reported.*

≥30% reduction in eGFR from baseline at 42 days

The proportion of participants with a ≥30% reduction in estimated glomerular filtration rate (eGFR) from baseline* that persists until 42 days after randomisation.

Participants that do not require a follow-up visit for the purposes of this core outcome measure:

a) Any participants that do not develop a ≥30% reduction in eGFR within 7 days of randomisation

b) Participants whose eGFR returns to within <30% of their baseline eGFR between randomisation and day 42 of follow-up (i.e., renal function has already recovered before 42 days).

**Where a baseline creatinine value is not available for a participant (from a previous blood test conducted in the year prior to the snakebite), the baseline eGFR should be assumed to be 100 ml/min/1.73 m^2.*

Hypotension:

Hypotensive shock at 3-hours

Proportion of participants with shock at 3-hours after randomization (shock defined as systolic blood pressure <90mmHg in adults, or age adjusted blood pressure in children*)

* Age adjusted systolic blood pressure threshold for defining hypotension:

**Age range**	**Systolic blood pressure cut-off for hypotension**
<12 months of age	<50 mm Hg
1-5 years of age	<60 mm Hg
6-12 years of age	<70 mm Hg
>13 years	<90 mm Hg

Overview of Core Outcome Measure Timepoints

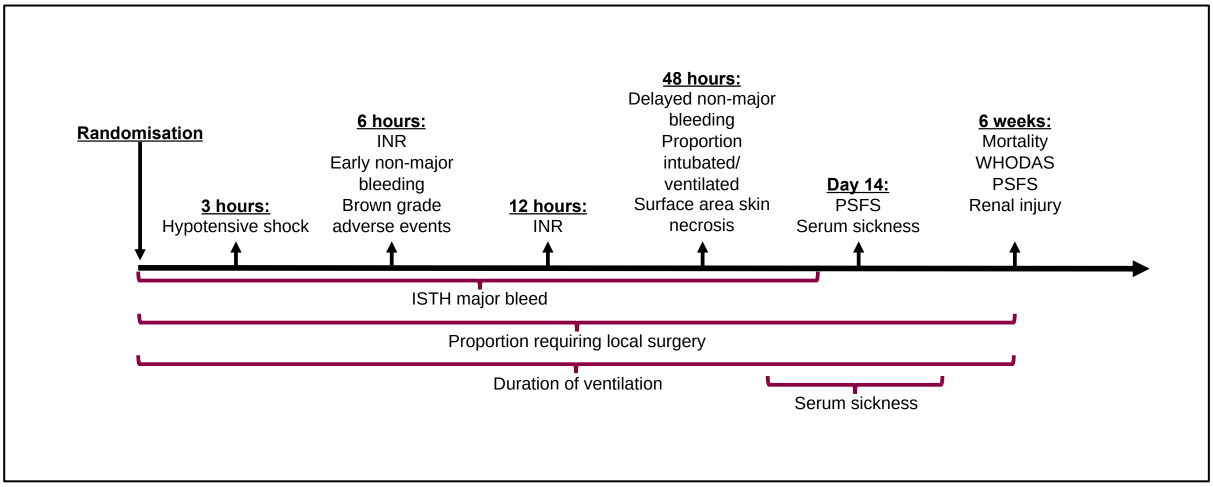

The Core Outcome Measurement Set Outcome Measurement Instruments and Definitions

12-item WHODAS 2.0 Outcome Measurement Instrument

This questionnaire asks about difficulties due to health conditions. Health conditions include diseases or illnesses, other health problems that may be short or long lasting, injuries, mental or emotional problems, and problems with alcohol or drugs.

Think back over the past 30 days and answer these questions, thinking about how much difficulty you had doing the following activities. For each question, please circle only one response.

In the past 30 days, how much difficulty did you have in:						
	**Score**	**1**	**2**	**3**	**4**	**5**
S1	Standing for long periods such as 30 minutes?	None	Mild	Moderate	Severe	Extreme or cannot do
S2	Taking care of your household responsibilities?	None	Mild	Moderate	Severe	Extreme or cannot do
S3	Learning a new task, for example, learning how to get to a new place?	None	Mild	Moderate	Severe	Extreme or cannot do
S4	How much of a problem did you have joining in community activities (for example, festivities, religious or other activities) in the same way as anyone else can?	None	Mild	Moderate	Severe	Extreme or cannot do
S5	How much have you been emotionally affected by your health problems?	None	Mild	Moderate	Severe	Extreme or cannot do
S6	Concentrating on doing something for ten minutes?	None	Mild	Moderate	Severe	Extreme or cannot do
S7	Walking a long distance such as a kilometre [or equivalent]?	None	Mild	Moderate	Severe	Extreme or cannot do
S8	Washing your whole body?	None	Mild	Moderate	Severe	Extreme or cannot do
S9	Getting dressed?	None	Mild	Moderate	Severe	Extreme or cannot do
S10	Dealing with people you do not know?	None	Mild	Moderate	Severe	Extreme or cannot do
S11	Maintaining a friendship?	None	Mild	Moderate	Severe	Extreme or cannot do
S12	Your day-to-day work?	None	Mild	Moderate	Severe	Extreme or cannot do

Total score: _____ / 60

H1	Overall, in the past 30 days, how many days were these difficulties present?	Record number of days ____
H2	In the past 30 days, for how many days were you totally unable to carry out your usual activities or work because of any health condition?	Record number of days ____
H3	In the past 30 days, not counting the days that you were totally unable, for how many days did you cut back or reduce your usual activities or work because of any health condition?	Record number of days ____

12-item WHODAS-child Outcome Measurement Instrument

In the past 30 days, how much difficulty did you have in:						
	**Score**	**1**	**2**	**3**	**4**	**5**
S1	Standing for a reasonable period of time, for example, in PE or school assembly or church/temple?	None	Mild	Moderate	Severe	Extreme or cannot do
S2	Doing chores or other things you are expected to do at home to help out?	None	Mild	Moderate	Severe	Extreme or cannot do
S3	Learning how to do something new, for example, how to play a new game, or learning something new at school?	None	Mild	Moderate	Severe	Extreme or cannot do
S4	Do you have more of a problem joining in community activities (for example, clubs, religious groups, or after-school activities) than you thought you should?	None	Mild	Moderate	Severe	Extreme or cannot do
S5	How much have you been upset by your health condition?	None	Mild	Moderate	Severe	Extreme or cannot do
S6	Concentrating for 10 minutes at a time or more while doing homework, playing a game, or doing something you were asked to do?	None	Mild	Moderate	Severe	Extreme or cannot do
S7	Walking for as long a distance as other people your age can?	None	Mild	Moderate	Severe	Extreme or cannot do
S8	Keeping yourself and your clothes clean, taking baths or showers, and brushing your teeth without being asked?	None	Mild	Moderate	Severe	Extreme or cannot do
S9	Getting dressed on your own?	None	Mild	Moderate	Severe	Extreme or cannot do
S10	Getting along with people you do not know well?	None	Mild	Moderate	Severe	Extreme or cannot do
S11	Keeping a friendship?	None	Mild	Moderate	Severe	Extreme or cannot do
S12	Doing your regular school assignments?	None	Mild	Moderate	Severe	Extreme or cannot do

Total score: _____ / 60

H1	Overall, in the past 30 days, how many days were these difficulties present?	Record number of days ____
H2	In the past 30 days, for how many days were you totally unable to carry out your usual activities or school/work because of any health condition?	Record number of days ____
H3	In the past 30 days, not counting the days that you were totally unable, for how many days did you cut back or reduce your usual activities or school/work because of any health condition?	Record number of days ____
H4	In the past 30 days, how many days were you absent from school?	Record number of days ____

Patient Specific Functional Scale Outcome Measurement Instrument

Read at baseline assessment: I am going to ask you to identify up to 3 important activities that you are unable to do or have difficulty with as a result of your problem.*

1)
2)
3)

Read at baseline and at follow-up assessments: Today, are there any activities that you are unable to do or have difficulty with because of your problem? (show scale)

For children aged <18 years, is this assessment being completed:

a) Independently by the child ☐

b) With support from a parent or caregiver ☐

c) By a parent or caregiver (with limited input from the child) ☐

Scale:

0	1	2	3	4	5	6	7	8	9	10
Unable to perform									Able to perform activity at pre-injury levels	

Score diary:

Activity	Date/score									
1										
2										
3										

For children, the parent or caregiver can assist the child with selecting the three activities. Alternatively, particularly for younger children, the parent or caregiver can identify activities that they are concerned about. For younger children, the parent or caregiver can rate the ability from 1-10 on behalf of the child. This should be a judgement decision based on whether the child is able to complete this assessment independently, if they need support, or if the assessment is more accurately completed by the parent or caregiver.

Brown Grading of Allergic Reactions Outcome Measurement Instrument

1) Mild (skin and subcutaneous tissues only)*	Generalized erythema, urticaria, periorbital oedema, or angioedema
2) Moderate (features suggesting respiratory, cardiovascular, or gastrointestinal involvement)	Dyspnoea, stridor, wheeze, nausea, vomiting, dizziness (presyncope), diaphoresis, chest or throat tightness,<br>or abdominal pain
3) Severe (hypoxia, hypotension, or neurologic compromise)	Cyanosis or SpO2 ≤92% at<br>any stage, hypotension* (SBP < 90mmHg in adults), confusion, collapse, LOC, or incontinence

Age Specific Definitions of Hypotensive Shock in Children

**Age range**	**Systolic blood pressure cut-off for hypotension**
<12 months of age	<50 mm Hg
1-5 years of age	<60 mm Hg
6-12 years of age	<70 mm Hg
>13 years	<90 mm Hg

Australian Snakebite Project (ASP) definition of serum sickness

≥3 of the following clinical features starting 5–20 days after antivenom administration:
• Fever
• Erythematous rash or urticaria
• Myalgia or arthralgia
• Arthritis
• Headache
• Malaise
• Nausea or vomiting

International Society on Thrombosis and Haemostasis (ISTH) Definition of Major Bleeding

• Fatal bleeding OR
• Symptomatic bleeding in a critical area or organ (such as intracranial, intraspinal, intraocular, retroperitoneal, intraarticular, pericardial, or intramuscular with compartment syndrome) OR
• Bleeding causing a fall in haemoglobin level of 2 g/dL (1.24 mmol/L) or more; or leading to transfusion of two or more units of whole blood or red cells.
